# Ropinirole and Pramipexole Promote Structural Plasticity in Human iPSC-Derived Dopaminergic Neurons via BDNF and mTOR Signaling

**DOI:** 10.1155/2018/4196961

**Published:** 2018-02-04

**Authors:** Ginetta Collo, Laura Cavalleri, Federica Bono, Cristina Mora, Stefania Fedele, Roberto William Invernizzi, Massimo Gennarelli, Giovanna Piovani, Tilo Kunath, Mark J. Millan, Emilio Merlo Pich, PierFranco Spano

**Affiliations:** ^1^Department of Molecular and Translational Medicine, University of Brescia, Brescia, Italy; ^2^Department of Biomedicine, University of Basel, Basel, Switzerland; ^3^Centro Salute e Ricerca “Camillo Golgi Salute della Donna”, University of Brescia, Brescia, Italy; ^4^IRCCS Istituto di Ricerche Farmacologiche “Mario Negri”, Milan, Italy; ^5^Section of Genetics, IRCCS “Centro S. Giovanni di Dio” Fatebenefratelli, Brescia, Italy; ^6^MRC Centre for Regenerative Medicine, Institute for Stem Cell Research, University of Edinburgh, Edinburgh, UK; ^7^Division of Psychopharmacology, Institut de Recherches Servier, Croissy-sur-Seine, France; ^8^CNS Therapeutic Area Unit, Takeda Development Center Europe, London, UK

## Abstract

The antiparkinsonian ropinirole and pramipexole are D3 receptor- (D3R-) preferring dopaminergic (DA) agonists used as adjunctive therapeutics for the treatment resistant depression (TRD). While the exact antidepressant mechanism of action remains uncertain, a role for D3R in the restoration of impaired neuroplasticity occurring in TRD has been proposed. Since D3R agonists are highly expressed on DA neurons in humans, we studied the effect of ropinirole and pramipexole on structural plasticity using a translational model of human-inducible pluripotent stem cells (hiPSCs). Two hiPSC clones from healthy donors were differentiated into midbrain DA neurons. Ropinirole and pramipexole produced dose-dependent increases of dendritic arborization and soma size after 3 days of culture, effects antagonized by the selective D3R antagonists SB277011-A and S33084 and by the mTOR pathway kinase inhibitors LY294002 and rapamycin. All treatments were also effective in attenuating the D3R-dependent increase of p70S6-kinase phosphorylation. Immunoneutralisation of BDNF, inhibition of TrkB receptors, and blockade of MEK-ERK signaling likewise prevented ropinirole-induced structural plasticity, suggesting a critical interaction between BDNF and D3R signaling pathways. The highly similar profiles of data acquired with DA neurons derived from two hiPSC clones underpin their reliability for characterization of pharmacological agents acting via dopaminergic mechanisms.

## 1. Introduction

Ropinirole and pramipexole are nonergoline dopaminergic agonists indicated for the treatment of Parkinson's disease and restless leg syndrome (RLS) [1, 2]. Improvement of depressive symptoms has also been consistently seen in these patients [3, 4], while controlled clinical trials demonstrated antidepressant efficacy mainly as adjunctive treatment in insufficiently responsive patients with mood disorders [5–8]. The latter observations are consistent with experimental data showing marked effects of these and other dopaminergic agonists in animal models of antidepressant properties [9–11].

Ropinirole and pramipexole behave as high efficacy agonists at D2 and D3 dopamine receptors (D2R and D3R), displaying a preference for D3R [2, 12, 13]. While a role of postsynaptic D2R in the *rapid* antidepressant actions of D2/D3 agonists has been demonstrated in experimental models [14], the significance of D3R sites remains less clear, in particular as regards to their *long-term* effects [9, 10]. The notion that D3R may fulfill a contrasting role compared with D2R is supported by differences in intracellular signaling cascades and fine control of dopaminergic transmission [15–18], as well as by their differential cerebral distribution, regulation, and functional segregation [19]. For example, in rodents, antagonism of D2R and D3R in the frontal cortex disrupts and promotes cognitive function, respectively [19–21].

Of particular interest are D3 autoreceptors expressed in DA neurons [22]: PET imaging studies in humans using D3R-selective ligands showed that the ventral mesencephalon expresses mainly if not uniquely D3R [19, 23]. A potential role of D3 autoreceptors in the actions of ropinirole and pramipexole is supported by two large imaging studies in Parkinson's patients: chronic treatment with either ropinirole or pramipexole revealed evidence for attenuation in the progressive reduction of DA neuron markers [24, 25]. Despite some methodological questions concerning the interpretation, these results are compatible with D3R-dependent neurorestorative effects associated with the preservation of DA terminals in surviving neurons, as experimentally shown in rodent models [26, 27]. In support of possible neurorestorative effects, we previously showed that D3R-preferential DA agonists increase dendrite arborization and soma size in cultured mouse mesencephalic DA neurons by activation of the mammalian target of rapamycin (mTOR) and extracellular signal-regulated kinase (ERK) [16, 28, 29], two molecular pathways critical for cell growth and structural remodeling [30]. This is of particular relevance to the *long-term* influence of ropinirole and pramipexole upon depression, in particular anhedonia, which is characterized in rodents by deficient dopaminergic transmission [31] and reduced neuroplasticity [32, 33].

One reservation with these and other studies performed in animal models is that they only partially recapitulate human cellular biology [34]. An alternative translational paradigm is offered by human-inducible pluripotent stem cells (hiPSCs) [35]. Several laboratories have developed protocols to differentiate hiPSCs into midbrain DA neurons [36, 37]. Over the last few years, this approach has been used for modeling CNS disorders, including rare monogenetic forms of Parkinson's disease [36, 38, 39]. Conversely, comparatively few studies have been dedicated to the pharmacology of antiparkinsonian drugs [38, 39] and the actions of ropinirole and pramipexole at hiPSCs differentiated into DA neurons have never, to our knowledge, been investigated.

In light of the abovementioned, the present work studied the putative role of D3R in the influence of ropinirole and pramipexole upon structural plasticity in midbrain DA neurons derived from hiPSCs. A high degree of coherence was found in the responses of two clones acquired from different donors. Both ropinirole and pramipexole increased dendritic arborization and soma size in human DA neurons via BDNF and mTOR signaling, underpinning observations in mouse mesencephalic DA neurons. These results support the relevance of D3 autoreceptors on dopaminergic neurons in the long-term neuroplastic actions of DA agonists and also the use of hiPSC-derived DA neurons as a translational model to study novel treatments for CNS disorders involving dopaminergic pathways.

## 2. Materials and Methods

### 2.1. Animals

CD1 mice were provided by Charles River Laboratories (Calco, Italy). Animal care was in accordance with the European Community Council Directive of September 2010 (2010/63/EU) with the approval of the Institutional Animal Care and Use Committee of the University of Brescia and in line with the Italian law. Mice breeding was performed to achieve timed pregnancy with the accuracy of ±0.5 days. The embryonic day (E) was determined by considering the day of insemination (determined by vaginal plug) as E0.5.

### 2.2. Pharmacological Agents

Ropinirole, pramipexole, pharmacological inhibitors, and receptor antagonists used in the present study are detailed in Supplementary Table S1. For each vehicle treatment, solvents required by specific drugs were used at the same dilution used for the active treatment.

### 2.3. Mouse Primary Mesencephalic Cultures

Primary mesencephalic cultures were prepared as previously described [16]. Ventral mesencephalon tissues were dissected from E12.5 CD1 mouse embryos and mechanically dissociated in Accumax (Sigma-Aldrich, Milan, Italy). Cells were counted and seeded on poly-D-lysine/laminin-coated cover slides (Sigma-Aldrich) (5 × 10^4^/ml) in Neurobasal medium (Gibco-Invitrogen, Carlsbad, CA) with the addiction of 2 mM glutamine (EuroClone) and B27 supplement (Gibco-Invitrogen). Cultures were maintained at 37°C in a humidified atmosphere of 5% CO_2_ and 95% air. Pharmacological treatments were conducted at least 5 days after seeding.

### 2.4. Human Fibroblasts

Dermal biopsies were obtained from healthy donors following the approval of the local ethics committee (CEIOC—Fatebenefratelli Hospital “San Giovanni di Dio,” Brescia, Italy, 44/2001 and 39/2005) and informed consent for use in research applications. CP01 fibroblasts from the dermal biopsy of a 40-year-old healthy Caucasian female donor were used at passage 3 for the reprogramming to hiPSCs. Fibroblasts were cultured in DMEM containing 10% FBS.

### 2.5. Human iPSC Generation

Lentivirus production and human fibroblast reprogramming to hiPSCs were performed according to Maherali et al. [40] (see Supplementary Materials (available here) and Methods). Methods for the characterization of hiPSCs are described in Supplementary Materials and Methods.

### 2.6. Dopaminergic Differentiation of Human iPSCs

**Figure 1:**
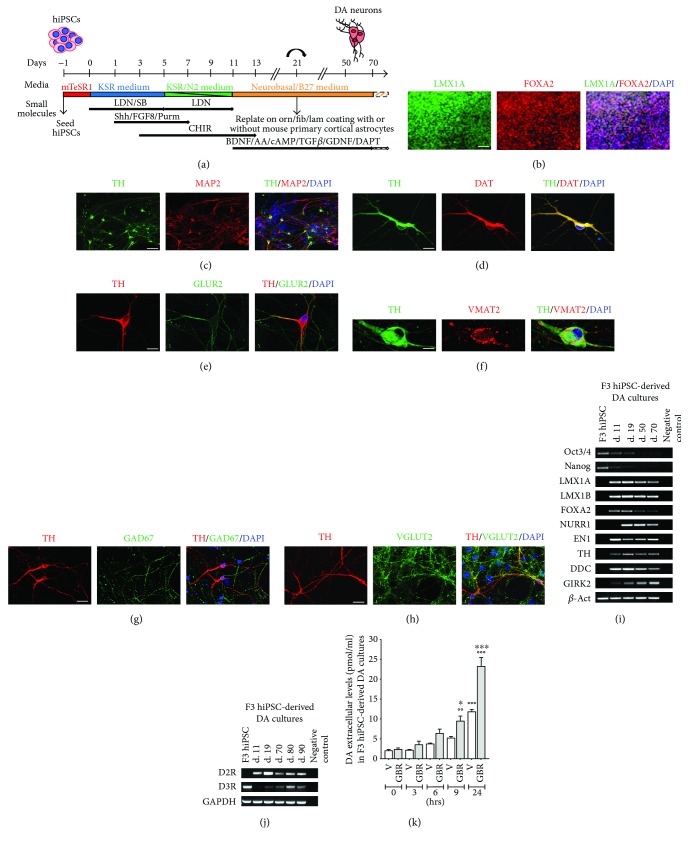
Differentiation of F3 human iPSCs in dopaminergic neurons. (a) Diagram of the time and conditions used for the differentiation of dopaminergic neurons from hiPSCs. (b–f) Representative images of dual immunofluorescence indicating coexpression of (b) LMX1-A (green) and FOXA2 (red) in dopaminergic progenitors at day 11; (c) TH (green) and MAP2 (red) in neurons at day 30; (d) TH (green) and DAT (red); (e) TH (red) and GLUR2 (green); and (f) TH (green) and VMAT2 (red) in DA neurons at day 70. (g, h) Dual immunofluorescence of (g) TH (red) and GAD67 (green) and (h) TH (red) and VGLUT2 (green) in neuronal cultures at day 70. Cell nuclei were stained with DAPI (blue). Scale bar: (b, c) = 40 *μ*m; (d, e, g, and h) = 20 *μ*m; and (f) = 10 *μ*m. (i, j) Semiquantitative RT-PCR analysis of (i) gene expression at the iPSC stage and at day 11, 19, 50, and 70 of dopaminergic differentiation and (j) expression of D2 and D3 receptors at the iPSC stage and at day 11, 19, 70, 80, and 90 of dopaminergic differentiation (negative controls contain PCR Master Mix and primers, but no cDNA). (k) Dopamine level measured by HPLC in the supernatant of F3 DA cultures (day 70) following incubation with the DAT inhibitor GBR12935 (GBR) or vehicle (V) over a time course (0, 3, 6, 9, and 24 hrs after administration). Data are expressed as mean ± SEM (^∗∗∗^ *p* < 0.001 versus vehicle at 24 hrs, ^∗^ *p* < 0.05 versus vehicle at 9 hrs, and °°°*p* < 0.001 and °°*p* < 0.01 versus the corresponding treatment (GBR or V) at time 0 h; post hoc Bonferroni's test). KSR, knockout serum replacement; LDN, LDN193189; SB, SB431542; Shh, Shh C25II; FGF8, fibroblast growth factor 8; Purm, purmorphamine; CHIR, CHIR99021; BDNF, brain-derived neurotrophic factor; AA, ascorbic acid; cAMP, dibutyryl cAMP; TGF*β*, transforming growth factor type *β*3; GDNF, glial cell line-derived neurotrophic factor.

Human iPSCs were induced to differentiate into floorplate- (FP-) derived midbrain DA neurons using dual SMAD inhibition and FP induction protocol [37] with minor modifications (Figure 1(a)). Human iPSCs were dissociated with Accutase™ (StemCell Technologies), seeded (3 × 10^4^ cells/cm^2^) on Matrigel-coated plates in Knockout Serum Replacement (KSR) medium containing Knockout™ DMEM, 15% KSR, GlutaMAX™, and 10 *μ*M 2-mercaptoethanol, in the presence of LDN193189 (0.1 *μ*M, Stemgent, Cambridge, MA), SB431542 (10 *μ*M, Tocris Bioscience, Bristol, UK), Shh C25II (0.1 *μ*g/ml, R&D Systems), Purmorphamine (2 *μ*M, Stemgent), fibroblast growth factor 8 (0.1 *μ*g/ml, R&D Systems), and CHIR99021 (3 *μ*M, Stemgent). From day 5, KSR medium was gradually shifted to N2 medium (Knockout DMEM/F12, N2 supplement, and GlutaMAX, all from Gibco-Invitrogen). On day 11, the medium was changed to Neurobasal/B27/GlutaMAX™ supplemented with CHIR99021, brain-derived neurotrophic factor (BDNF; 20 ng/ml, R&D Systems), ascorbic acid (AA; 0.2 mM, Sigma-Aldrich), dibutyryl cAMP (cAMP; 0.5 mM, Sigma-Aldrich), transforming growth factor type *β*3 (TGF*β*3; 1 ng/ml, R&D Systems), glial cell line-derived neurotrophic factor (GDNF; 20 ng/ml, R&D Systems), and DAPT (10 nM, Tocris Bioscience). On day 21, cells were dissociated and seeded (5 × 10^4^ cells/cm^2^) on plates precoated with polyornithine (15 *μ*g/ml), fibronectin (2 *μ*g/ml), and laminin (1 *μ*g/ml) (all from Sigma-Aldrich) and cultured in the same medium until day 70, when they were used for biochemical studies. For morphological studies, on day 21, cells were seeded on plates precoated with polyornithine/fibronectin/laminin and cocultured with mouse primary cortical astrocytes [41] that were isolated and cultured according to Sorg and Magistretti [42]. Human iPSC-derived DA neurons were used for pharmacological studies, immunofluorescence, immunocytochemistry, and morphological analysis starting from day 70. Three days before pharmacological treatments, BDNF, AA, cAMP, TGF*β*3, GDNF, and DAPT were gradually removed from the culture. Criteria to define DA neurons at day 70 were the following: neuronal morphology, coexpression of TH/MAP2, coexpression of TH/dopamine transporter (DAT), coexpression of TH/vesicular monoamine-associated transporter 2 (VMAT2), coexpression of TH/AMPAR subunit GluR2, and functional dopamine release and uptake from cultures containing DA neurons.

### 2.7. *In Vitro* Pharmacological Experiments

All pharmacological treatments were performed from day 70 in culture. For morphological studies, neuronal cultures were exposed 72 hrs to the DA agonists ropinirole or pramipexole. Pharmacological inhibitors and receptor antagonists were added to the cultures 20 min prior treatment with DA agonists, and cultures were fixed at the end of the treatment. For Western blot and immunofluorescence analysis of p70S6K phosphorylation, cultures were exposed to DA agonists for periods ranging from 2 to 60 min. For inhibition experiments, pharmacological inhibitors and receptor antagonists were added to the cultures 20 min prior treatment with DA agonists and analyses were performed 2 min after exposure.

### 2.8. Immunofluorescence and Immunocytochemistry

Immunofluorescence of hiPSCs, embryoid bodies (EBs), and hiPSC-derived DA neurons is described in Supplementary Materials and Methods. Immunocytochemistry of DA neurons for morphological analysis was performed using anti-tyrosine hydroxylase (TH) rabbit polyclonal antibody (pAb; Santa Cruz Biotechnology, Santa Cruz, CA) (Supplementary Table S2), followed by incubation with a biotinylated goat anti-rabbit antibody (Jackson ImmunoResearch) (Supplementary Table S3), as previously described [16, 28]. Immunofluorescence quantification of p-p70S6K in dopaminergic (TH^+^/MAP2^+^) and nondopaminergic (TH^−^/MAP2^+^) neurons was performed using ImageJ 2.0.0–rc-59/1.51k software. Measures of pixel intensity and area with respect to background intensity and cell surface, respectively, were calculated according to the following formula: (intensity of sample stained cell/area of sample stained cell) − (intensity of the sample of background/area of sample background). To minimize the inherent variability in the immunofluorescence procedure, cover slides from the same experiment were processed simultaneously.

### 2.9. Computer-Assisted Morphological Analysis

Digital images were acquired with an Olympus IX51 microscope connected to an Olympus (Hamburg, Germany) digital camera and a PC. Morphometric measurements were performed by a blinded examiner on digitalized images using Image-Pro Plus software (Media Cybernetics, Bethesda, MD). Morphological indicators of structural plasticity were considered: (i) the maximal dendrite length, (ii) the number of primary dendrites, and (iii) the soma area [16]. The maximal dendrite length was defined as the distance from the soma (hillock base) to the tip of the longest dendrite for each neuron; dendrites shorter than 20 *μ*m were excluded from the analysis. Primary dendrites were defined as those directly stemming from the soma. The soma area was assessed by measuring the surface (*μ*m^2^) included by the external perimeter drawn on the cell membrane of neurons identified by TH^+^ staining. Two cover slides per treatment groups were examined to obtain measurements from at least 30–50 neurons. Each experiment was repeated three times.

### 2.10. Western Blotting

Western blotting was performed as previously described [28], at different time points (2–60 min) following challenge with ropinirole (10 *μ*M) and/or after pretreatments with either D3R antagonists SB277011-A (100 nM), S33084 (10 nM), MEK inhibitor PD98059 (10 *μ*M), or PI3-K inhibitor LY294002 (10 *μ*M). Primary antibodies used were the following: anti-p-p70S6K mouse monoclonal antibody (mAb), anti-p70S6K rabbit mAb (all from Cell Signaling Technology, Danvers, MA), anti-TH rabbit pAb (Merck Millipore), and anti-*α*-Tubulin mouse mAb (Sigma-Aldrich) (Supplementary Table S2). In each experiment, the same membrane was processed in the following order: incubation with anti-p-p70S6K antibody, incubation with anti-p70S6K antibody, stripping with the Re-Blot Plus Strong Solution (Merck Millipore), incubation with anti-TH antibody, and final incubation with anti-*α*-Tubulin antibody. After the incubation with primary antibodies, blots were incubated with appropriate horseradish peroxidase-conjugated secondary antibodies (Santa Cruz Biotechnology) (Supplementary Table S3) and developed using a chemiluminescent substrate (ECL, LiteAblot Extend; EuroClone). Specific bands were analyzed by densitometric scanning of the exposed film using Gel-Pro analyzer software (Media Cybernetics). In each experiment, the specific signal of p-p70S6K protein was normalised to the corresponding p70S6K signal and then to the level of TH and *α*-Tubulin measured in the same preparation.

### 2.11. Measurement of Dopamine Release

The dopamine released in the culture medium was determined by the HPLC method coupled with electrochemical detection used for the assay of dopamine in rodent brain with minor modifications [43]. Human iPSC-derived DA neurons cultured for 70 days were incubated with either vehicle or the dopamine reuptake inhibitor GBR12935 (30 nM) (Sigma-Aldrich) up to 24 hrs. Fractions of medium were collected at different time points, that is, at 0, 3, 6, 9, and 24 hrs after incubation. 2 M HClO_4_ containing 0.5% Na_2_S_2_O_5_ and 1% Na_2_EDTA (all from Sigma-Aldrich) was diluted at 1 : 20 in each fraction. Samples were kept at −80°C until HPLC assay. HPLC consisted of a constant flow LC20-AD pump (Shimadzu, Italy) and a Coulochem II electrochemical detector equipped with a dual electrode 5011 analytical cell (ESA, Chelmsford, MA). Potential settings were E1‐175 mV and E2‐300 mV. Twenty-five microliter samples were injected into the HPLC with a refrigerated (5°C) autosampler (Midas, Spark Holland, The Netherlands). Dopamine was separated through an Accucore XL C18 column, 150 × 3 mm, particle size 4 *μ*m (Thermo Fisher Scientific) protected with a guard column (NewGuard RP-18, 7 *μ*m, 15 × 3.2 mm; Perkin Elmer, Italy). The column was maintained at 40°C. Mobile phase consisted of 5 g/l anhydrous CH_3_COONa, 3.57 g/l citric acid, 112 mg/l Na_2_EDTA, 200 mg/l sodium octane sulfate, and 70 ml/l CH_3_OH. Flow rate was 0.5 ml/min. Assay was calibrated daily by injecting 2.5, 25, and 250 fmol/25 *μ*l DA made up in HClO_4_ 0.2 M plus 0.05% Na_2_S_2_O_5_ and 0.1% Na_2_EDTA. The detection limit was 2 fmol on the column (signal-to-noise ratio 2).

### 2.12. RNA Extraction and RT-PCR Analysis

RNA extraction and RT-PCR analysis are described in Supplementary Materials and Methods. Sequences of individual primer pairs and melting temperature (Tm) are detailed in Supplementary Table S4.

### 2.13. Statistical Analysis

Data were expressed as mean ± standard error of the mean (SEM) if not stated otherwise. Significant differences from control conditions were determined using either one-way or two-way analysis of variance (ANOVA) followed by posteriori Bonferroni's test for multiple comparisons provided by GraphPad Prism, version 6.0 software package (GraphPad Software, San Diego, CA).

## 3. Results

### 3.1. Generation of Human iPSCs and Differentiation into Mature DA Neurons

CP01 human fibroblasts from a healthy donor were reprogrammed according to the protocol of Maherali et al. [40] (Supplementary Figure S1A), resulting in several hiPSC colonies, including a clone called F3. In the present study, F3 hiPSCs were fully characterized: details are described in Supplementary Results and in Supplementary Figures S1 and S2. F3 and NAS2, previously published hiPSC clones from a healthy donor [36], were differentiated into midbrain DA neurons via floor plate induction as published by Kriks et al. [37] with minor modifications (Figure 1(a)). Dopaminergic differentiation of F3 hiPSCs is shown in Figures 1(b)–1(j). At day 11, immunofluorescence analysis of F3 midbrain floor plate precursors showed the distinctive coexpression of the floor plate marker FOXA2 and the roof plate marker LMX1-A [37]. At day 21, cells were plated on a mouse astrocyte feeder layer, and at day 30, MAP2^+^-TH^+^ neurons were present. At day 70, most TH^+^ neurons consistently coexpressed the dopamine transporter (DAT), the AMPAR subunit GluR2, and vesicular monoamine-associated transporter 2 (VMAT2), indicating a mature DA neuronal phenotype (Figures 1(b)–1(f)). GABAergic and glutamatergic neurons identified by expression of GAD67 and VGLUT2, respectively, were also present in the cultures (Figures 1(g) and 1(h)). Cell count indicated 30% ± 5% of TH^+^ neurons, 23% ± 4% of GAD67^+^ neurons, and 28% ± 6% of VGLUT2^+^ neurons costained with anti-MAP2 antibody. Semiquantitative RT-PCR analysis confirmed the expression of LMX1-A, LMX1-B, FOXA2, ENGRAILED 1 (EN1), TH and Dopa decarboxylase (DDC), G protein-coupled inwardly rectifying potassium channel (GIRK2) from day 11, and NURR1 from day 19. The mRNAs for Oct3/4 and Nanog, markers of pluripotency, progressively decreased during differentiation (Figure 1(i)). D2R mRNA was detectable from day 11 of differentiation, while D3R mRNA was expressed at the iPSC stage, not detectable at day 11 of differentiation while it started to appear again from day 19 (Figure 1(j)). Dopaminergic differentiation using NAS2 hiPSCs is represented in Supplementary Figure S3.

The neurochemical evidence of a functional DAT uptake of DA neurons cultured for 70 days was obtained by measuring the level of dopamine in the culture supernatant by HPLC following incubation with the DAT inhibitor GBR12935 or vehicle over a time course up to 24 hrs (Figure 1(k)). Two-way ANOVA showed a highly significant treatment effect (*F*_(1,20)_ = 47, *p* < 0.0001), time effect (*F*_(4,20)_ = 91, *p* < 0.0001), and interaction effect (*F*_(4,20)_ = 11, *p* < 0.0001), indicating a time-dependent pharmacological inhibition of DAT functions. These results indicate spontaneous release of dopamine, as shown by the progressive increase of dopamine levels after vehicle over time, and an active uptake of extracellular dopamine as shown by the strong increase of dopamine levels after GBR12935, as expected by mature DA neurons.

### 3.2. Effects of Ropinirole on Structural Plasticity of hiPSC-Derived and Mouse Mesencephalic DA Neurons

**Figure 2:**
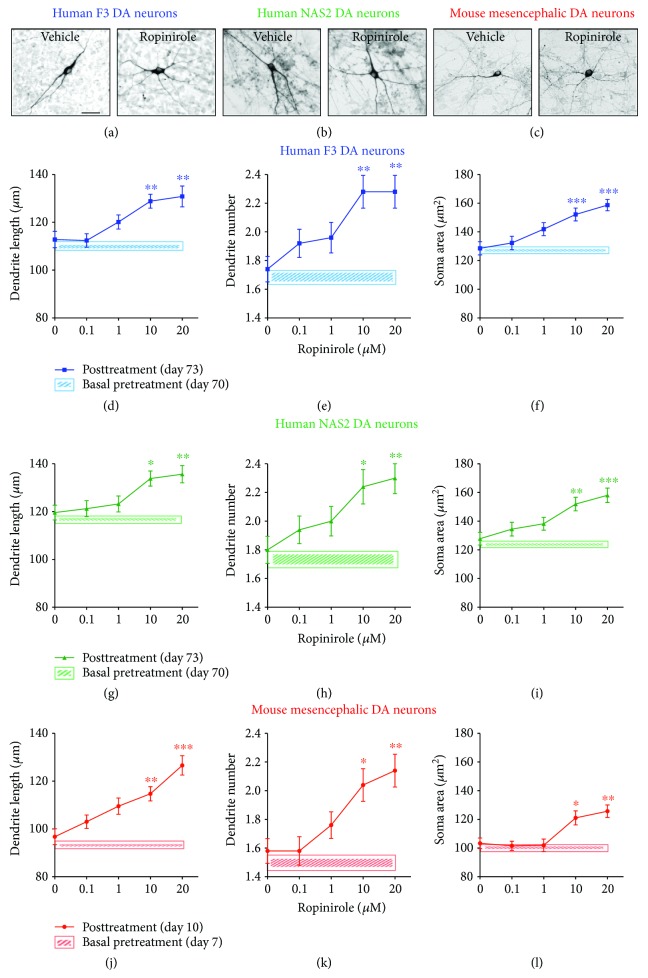
Ropinirole promotes structural plasticity of human iPSC-derived and mouse mesencephalic DA neurons. (a–c) Representative photomicrographs of human F3, human NAS2 (70 days in culture), and mouse mesencephalic DA neurons (7 days in culture) 72 hrs after exposure to vehicle or to 10 *μ*M ropinirole. Scale bar: 30 *μ*m. (d–l) Concentration-response curves of the effect of ropinirole on maximal length of dendrites, number of primary dendrites, and soma area of (d–f) human F3 DA neurons, (g–i) human NAS2 DA neurons, and (j–l) mouse mesencephalic DA neurons. Shaded areas: (d–f) light blue, (g–i) light green, and (j–l) light red show no significant changes in structural plasticity between basal measurements and measurements after 72 hrs when cultures were exposed to vehicle. Data are expressed as mean ± SEM (^∗∗∗^ *p* < 0.001, ^∗∗^ *p* < 0.01, and ^∗^ *p* < 0.05 versus vehicle (0); post hoc Bonferroni's test).

DA neurons derived from F3 and NAS2 hiPSCs were exposed to ropinirole for 72 hrs. In parallel experiments, primary cultures of mouse mesencephalic DA neurons known to respond to D3-preferential DA agonists [16], were also tested for 72 hrs (Figures 2(a)–2(l)). No changes in structural plasticity were observed between basal measurements and measurements after 72 hrs when cultures were exposed to vehicle (Figures 2(d)–2(l), shaded areas). Conversely, ropinirole (0.1–20 *μ*M) produced a dose-dependent effect on structural plasticity 72 hrs after the beginning of treatment (Figures 2(d)–2(l)). Two-way ANOVA indicated a significant treatment main effect for the maximal length of dendrites [*F*_(4,435)_ = 21.86, *p* < 0.0001], number of primary dendrites [*F*_(4,735)_ = 17.96, *p* < 0.0001], and soma area [*F*_(4,585)_ = 22.21, *p* < 0.0001], as well as a significant main effect of DA neuron type (i.e., F3 or NAS2 or mouse) for the maximal length of dendrites [*F*_(2,435)_ = 31.94, *p* < 0.0001], number of primary dendrites [*F*_(2,735)_ = 3.89, *p* < 0.05], and soma area [*F*_(2,585)_ = 84.75, *p* < 0.0001], while interaction was never significant. Within each culture, post hoc multiple comparisons indicated that the treatment effect versus vehicle started at the dose of 10 *μ*M (*p* < 0.05 or lower); no difference was observed between F3 and NAS2, while mouse mesencephalic DA neurons were consistently smaller than human DA neurons (*p* < 0.05 or lower, Bonferroni's test).

### 3.3. Rapid Activation of mTOR Pathway Induced by Ropinirole Is Mediated through D3R Signaling

**Figure 3:**
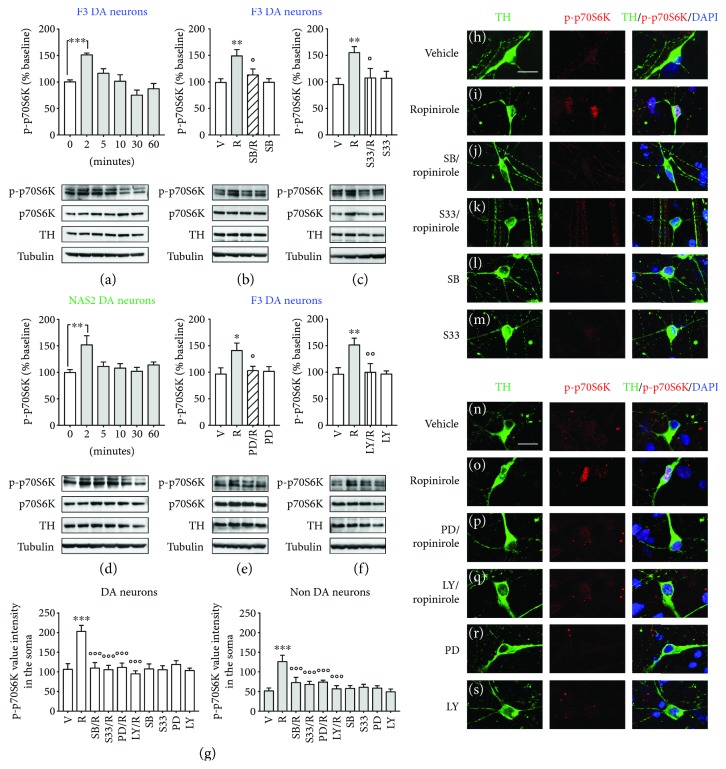
Ropinirole increases phosphorylation of p70S6K in human iPSC-derived DA neurons via activation of D3 receptor. (a, d) Time course of phosphorylated p70S6K (p-p70S6K) induced by ropinirole (10 *μ*M) in (a) F3 and (d) NAS2 DA cultures, respectively, at 0, 2, 5, 10, 30, and 60 min and analysed by densitometry of Western blots (*n* = 6) (top panels); the specific levels of p-p70S6K were normalised to the corresponding p70S6K, TH, and tubulin levels. The densitometric values are represented as the percentage of vehicle values. (lower panels) Representative Western blots. (b, c) Blockade of p-p70S6K induced by ropinirole (10 *μ*M) in F3 DA neurons after pretreatment (20 min) with the D3R antagonists (b) SB277011-A (100 nM) or (c) S33084 (10 nM) assessed after 2 min and analyzed by densitometry of Western blots (*n* = 3) (top panels). (lower panels) Representative Western blots. (h–m) Representative photomicrographs of F3 DA neurons showing p-p70S6K increase in the soma and dendrites of TH^+^ neurons 2 min after ropinirole (10 *μ*M) following pretreatment with vehicle (i). (j) Blockade of p-p70S6K by pretreatment (20 min) with either SB277011-A (100 nM) or (k) S33084 (10 nM) followed by ropinirole (2 min). (h) Vehicle; (l) SB277011-A alone; and (m) S33084 alone. TH (green) and p-p70S6K (red). Cell nuclei were stained with DAPI (blue). (e, f) Blockade of p-p70S6K induced by ropinirole (10 *μ*M) in F3 DA neurons after pretreatment (20 min) with (e) the MEK inhibitor PD98059 (10 *μ*M) or (f) the PI3-K inhibitor LY294002 (10 *μ*M) assessed after 2 min and analyzed by densitometry of Western blots (*n* = 3) (top panels). (lower panels) Representative Western blots. (n–s) Representative photomicrographs of F3 DA neurons showing p-p70S6K increase in the soma and dendrites of TH^+^ neurons 2 min after ropinirole (10 *μ*M) following pretreatment with vehicle (o). (p) Blockade of p-p70S6K by pretreatment (20 min) with PD98059 (10 *μ*M) or (q) LY294002 (10 *μ*M) followed by ropinirole (2 min). (n) Vehicle; (r) PD98059 alone; and (s) LY294002 alone. TH (green) and p-p70S6K (red). Cell nuclei were stained with DAPI (blue). Scale bar: 20 *μ*m. (g) Semiquantitative image analysis of p-p70S6K fluorescence intensity in TH^+^ and TH^−^ neurons. All data are expressed as mean values ± SEM (^∗∗∗^ *p* < 0.001, ^∗∗^ *p* < 0.01, and ^∗^ *p* < 0.05 versus vehicle; ^ooo^ *p* < 0.001, ^oo^ *p* < 0.01, and ^o^ *p* < 0.05 versus ropinirole; post hoc Bonferroni's test). V, vehicle; R, ropinirole; SB, SB277011-A; S33, S33084; PD, PD98059; LY, LY294002.

D3Rs are known to activate the intracellular pathway of MEK-ERK [15, 16] and PI3K-Akt [28, 29], leading to a mTOR-dependent increase of phosphorylated p70S6K (p-p70S6K) [28, 30]. The effect of ropinirole (10 *μ*M) on p-p70S6K in hiPSC-derived DA neurons was investigated by Western blot and immunofluorescence analysis. In acute experiments, using Western blot quantification, ropinirole significantly increased p-p70S6K after 2 min in both F3 and NAS2 hiPSC-derived DA cultures (Figures 3(a) and 3(d)), as supported by the significant main effect of time [*F*_(5,28)_ = 14.00, *p* < 0.0001] and the nonsignificant main effect of DA neuron type and interaction. The role of D3R was investigated by pretreatments with the selective D3R antagonists SB277011-A (100 nM) [44] and S33084 (10 nM) [45]. Both antagonists blocked the effect of ropinirole on p-p70S6K as measured by Western blot in F3 cultures (Figures 3(b) and 3(c)) and NAS2 cultures (Supplementary Figure S4A and B) at 2 min after exposure. Two-way ANOVA indicated the significant treatment main effect of SB277011-A and S33084 [*F*_(2,42)_ = 13.36, *p* < 0.0001] and [*F*_(2,42)_ = 17.44, *p* < 0.0001], respectively, while no significant DA neuron type main effect and interactions were observed. The potential involvement of MEK-ERK and PI3K-Akt pathways in mediating the effects of ropinirole on p-p70S6K was studied using a MEK inhibitor PD98059 (10 *μ*M) and a PI3-K inhibitor LY294002 (10 *μ*M). Pretreatment with both inhibitors separately blocked the effects of ropinirole measured in F3 cultures (Figures 3(e) and 3(f)) and NAS2 cultures (Supplementary Figure S4I and J). Two-way ANOVA of Western blots showed a significant treatment effect of PD98059 and LY294002 [*F*_(2,42)_ = 14.05, *p* < 0.0001] and [*F*_(2,27)_ = 10.90, *p* < 0.0001], respectively, while no significant DA neuron type effect or interaction was observed. No effects on p-p70S6K were observed when SB277011-A, S33084, PD98059, or LY294002 was incubated with vehicle.

Parallel immunofluorescence experiments of F3 and NAS2 hiPSC-derived DA cultures showed that ropinirole significantly increased the levels of p-p70S6K in TH^+^ neurons (Figures 3(i) and 3(o); Supplementary Figure S4D and L), partially accounting for the increases observed in the Western blot studies. In addition, ropinirole increased p-p70S6K in TH^−^ neurons, as expected since D3Rs are expressed also in non DA neurons [22]. Pretreatments with either SB277011-A or S33084 prevented the increase of p-p70S6K in TH^+^ as well as TH^−^ neurons following exposure to ropinirole in both F3 cultures (Figures 3(j) and 3(k)) and NAS2 cultures (Supplementary Figure S4E and F). Pretreatment with the MEK inhibitor PD98059 or the PI3-K inhibitor LY294002 blocked the effects of ropinirole measured in F3 cultures (Figures 3(p) and 3(q)) and NAS2 cultures (Supplementary Figure S4M and N). No effects on p-p70S6K were observed when SB277011-A, S33084, PD98059, or LY294002 was incubated with vehicle (Figures 3(l), 3(m), 3(r), and 3(s); Supplementary Figure S4G, H, O, and P). These observations were supported by the results of semiquantitative image analysis of p-p70S6K fluorescence intensity in TH^+^ neurons [two-way ANOVA interaction: *F*_(4,240)_ = 8.8, *p* < 0.0001; treatment factor: *F*_(4,240)_ = 8.4, *p* < 0.001; and inhibition factor: *F*_(1,240)_ = 5.9, *p* < 0.02] and in TH^−^ neurons [two-way ANOVA interaction: *F*_(4,240)_ = 5.9, *p* < 0.0002; treatment factor: *F*_(4,240)_ = 4.9, *p* < 0.001; and inhibition factor: *F*_(1,240)_ = 20.5, *p* < 0.0001] (Figure 3(g)).

### 3.4. Structural Plasticity Induced by Ropinirole Depends on mTOR-Mediated D3R Signaling

The role of D3R-dependent signaling in structural plasticity produced by ropinirole in human DA neurons was investigated 72 hrs after either the pharmacological blockade of D3R or the inhibition of the intracellular pathways leading to mTOR activation.

**Figure 4:**
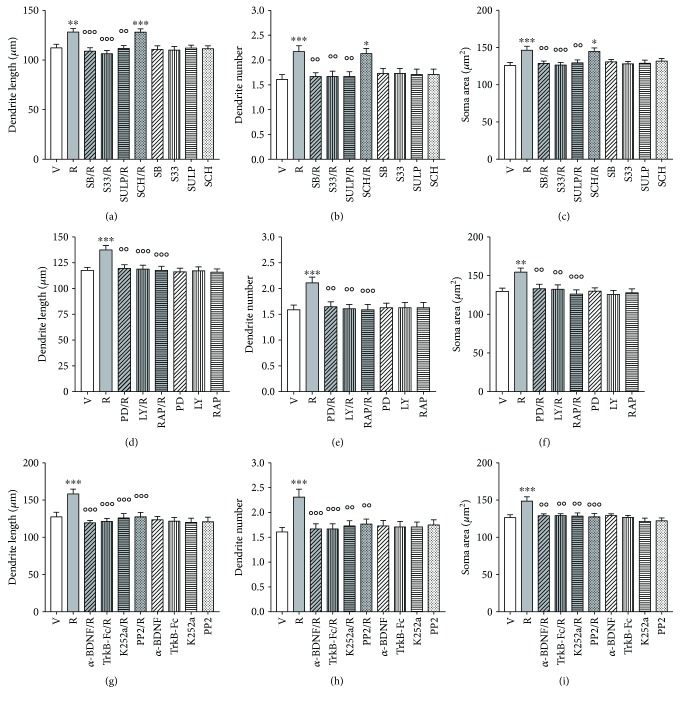
Structural plasticity induced by ropinirole in human iPSC-derived DA neurons is prevented by D3R antagonists, by MEK-ERK and PI3K-mTOR inhibitors and by BDNF-TrkB signaling inhibitors. (a–c) Inhibition of the effects of ropinirole (10 *μ*M) on structural plasticity of F3 DA neurons following pretreatment (20 min) with the D3R antagonists SB277011-A (50 nM) and S33084 (10 nM) or the D2/D3R antagonist sulpiride (5 *μ*M) assessed as (a) maximal dendrite length, (b) number of primary dendrites, and (c) soma area after 72 hrs. Pretreatment (20 min) with the D1R antagonist SCH23390 (1 *μ*M) was ineffective. (d–f) Inhibition of the effects of ropinirole (10 *μ*M) on structural plasticity of F3 DA neurons following pretreatment (20 min) with PD98059 (10 *μ*M), LY294002 (10 *μ*M), or rapamycin (20 nM), assessed as (d) maximal dendrite length, (e) number of primary dendrites, and (f) soma area after 72 hrs. (g–i) Inhibition of the effects of ropinirole (10 *μ*M) on structural plasticity of F3 DA neurons following pretreatment (20 min) with an anti-BDNF blocking antibody (*α*-BDNF) (10 *μ*g/ml), a TrkB-Fc Chimera (TrkB-Fc) (5 *μ*g/ml), the TrkB-phosphorylation inhibitor K252a (200 nM), and the TrkB-Src phosphorylation inhibitor PP2 (10 *μ*M), assessed as (g) maximal dendrite length, (h) number of primary dendrites, and (i) soma area after 72 hrs. When antagonists and inhibitors were tested with the vehicle, no changes of structural plasticity were visualized. In all panels, values are represented as mean ± SEM (^∗∗∗^ *p* < 0.001, ^∗∗^ *p* < 0.01, and ^∗^ *p* < 0.05 versus vehicle; °°°*p* < 0.001 and °°*p* < 0.01 versus ropinirole; post hoc Bonferroni's test). V, vehicle; R, ropinirole; SB, SB277011-A; S33, S33084; SULP, sulpiride; SCH, SCH23390; PD, PD98059; LY, LY294002; RAP, rapamycin, *α*-BDNF, anti-BDNF blocking antibody; TrkB-Fc, TrkB-Fc Chimera.

The effects of ropinirole versus vehicle on F3 hiPSC-derived DA neurons were significantly (*p* < 0.01, Bonferroni's test) attenuated by pretreatment with SB277011-A (50 nM), S33084 (10 nM), and the nonselective D2/D3R antagonist sulpiride (5 *μ*M), but not by the D1R antagonist SCH23390 (1 *μ*M) (Figures 4(a)–4(c)), as supported by the significant two-way ANOVA interaction obtained on the maximal length of dendrites [*F*_(4,290)_ = 5.4, *p* < 0.0001], number of primary dendrites [*F*_(4,490)_ = 4.9, *p* < 0.0001], and soma area [*F*_(4,390)_ = 4.3, *p* < 0.001]. No changes were seen when DA antagonists were dosed in the presence of vehicle.

The effects of ropinirole on structural plasticity were prevented by inhibition of the intracellular pathways using PD98059 (10 *μ*M), LY294002 (10 *μ*M), and the mTORC1 inhibitor rapamycin (20 nM). All kinase inhibitors significantly (*p* < 0.01, Bonferroni's test) counteracted the effects of ropinirole on all three structural plasticity parameters (Figures 4(d)–4(f)), as supported by the significant two-way ANOVA interaction obtained on the maximal length of dendrites [*F*_(3,232)_ = 11.0, *p* < 0.0001], number of primary dendrites [*F*_(3,392)_ = 6.6, *p* < 0.0001], and soma area [*F*_(3,312)_ = 5.1, *p* < 0.001]. No changes were seen when the kinase inhibitors were dosed in the presence of vehicle. Full tabular results of ANOVA are reported in Table 1.

Similar results were obtained in parallel experiments testing NAS2 DA neurons with DA antagonists (Supplementary Figure S5A–C) and kinase inhibitors (Supplementary Figure S5D–F), as supported by statistical analysis (Supplementary Table S5).

### 3.5. BDNF-TrkB Signaling Is Involved in Structural Plasticity Induced by Ropinirole

Four different approaches were used to block BDNF-TrkB signaling: immunoneutralization of extracellular BDNF using an anti-BDNF blocking antibody (*α*-BDNF), scavenging of extracellular BDNF using a TrkB-Fc Chimera, blockade of TrkB receptor using K252a [46], and inhibition of TrkB dependent Src phosphorylation using PP2. Pretreatments with all BDNF-TrkB signaling inhibitors antagonized the effects of ropinirole on all three structural plasticity parameters in both F3 DA neurons (Figures 4(g)–4(i)) and NAS2 DA neurons (Supplementary Figure S5G–I), as supported by the significant two-way ANOVA interaction obtained on the maximal length of dendrites [*F*_(4,290)_ = 12.0, *p* < 0.0001], number of primary dendrites [*F*_(4,490)_ = 3.5, *p* < 0.001], and soma area [*F*_(4,390)_ = 7.0, *p* < 0.0001]. No changes were seen when BDNF-TrkB signaling blockers were dosed in the presence of vehicle. Full tabular results of ANOVA are reported in Table 1.

Similar results were obtained in parallel experiments testing NAS2 DA neurons with BDNF-TrkB signaling inhibitors (Supplementary Figure S5G–I), as supported by statistical analysis (Supplementary Table S5).

### 3.6. Effects of Pramipexole on Structural Plasticity of hiPSC-Derived DA Neurons

**Figure 5:**
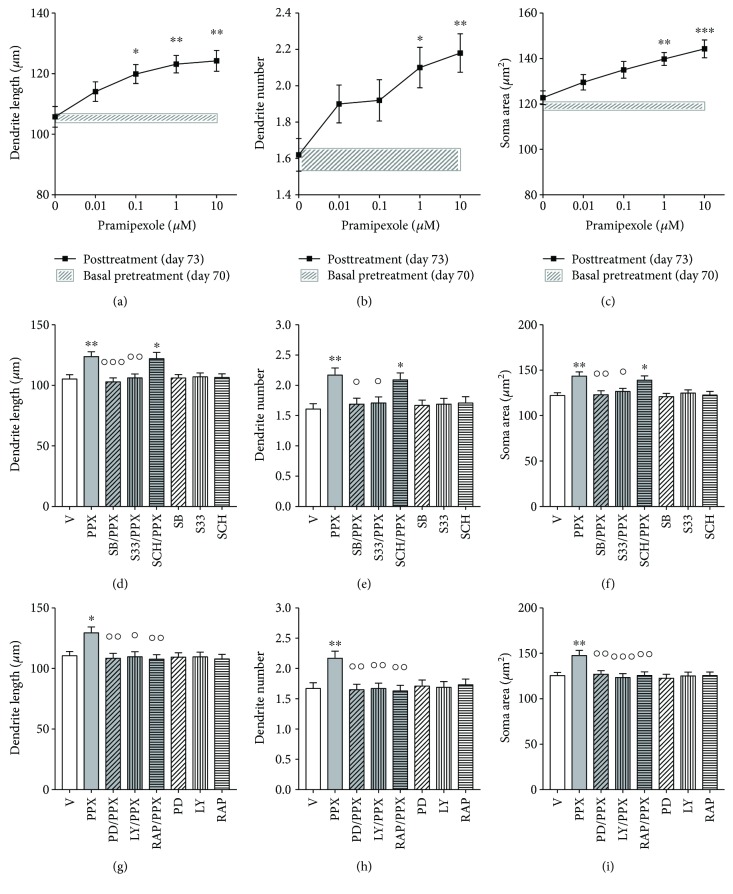
Structural plasticity induced by pramipexole in human iPSC-derived DA neurons is prevented by D3R antagonists and by MEK-ERK and PI3K-mTOR inhibitors. (a–c) Concentration-response curves of pramipexole effects on (a) maximal length of dendrites, (b) number of primary dendrites, and (c) soma area of human F3 DA neurons. Shaded areas (a–c), light grey, show no significant changes in structural plasticity between basal measurements and measurements after 72 hrs when cultures were exposed to vehicle. Data are expressed as mean ± SEM (^∗∗∗^ *p* < 0.001, ^∗∗^ *p* < 0.01, and ^∗^ *p* < 0.05 versus vehicle (0); post hoc Bonferroni's test). (d–f) Inhibition of the effects of pramipexole (10 *μ*M) on structural plasticity following pretreatment (20 min) with either SB277011-A (50 nM) or S33084 (10 nM); (d) maximal dendrite length, (e) number of primary dendrites, and (f) soma area after 72 hrs. Pretreatment (20 min) with SCH23390 (1 *μ*M) was ineffective. (g–i) Inhibition of the effects of pramipexole (10 *μ*M) on structural plasticity following pretreatment (20 min) with PD98059 (10 *μ*M), LY294002 (10 *μ*M), or rapamycin (20 nM); (g) maximal dendrite length, (h) number of primary dendrites, and (i) soma area after 72 hrs. No changes of structural plasticity were visualized when antagonists and inhibitors were tested with the vehicle. Data are expressed as mean ± SEM (^∗∗∗^ *p* < 0.001, ^∗∗^ *p* < 0.01, and ^∗^ *p* < 0.05 versus vehicle; °°°*p* < 0.001, °°*p* < 0.01, and °*p* < 0.05 versus pramipexole; post hoc Bonferroni's test). V, vehicle; PPX, pramipexole; SB, SB277011-A; S33, S33084; SCH, SCH23390; PD, PD98059; LY, LY294002; RAP, rapamycin.

Pramipexole (0.01–10 *μ*M) produced a dose-dependent effect on structural plasticity of F3 DA neurons 72 hrs after the beginning of treatment. One-way ANOVA indicated a significant dose-dependent effect for the maximal length of dendrites [*F*_(4,145)_ = 5.57, *p* < 0.001], number of primary dendrites [*F*_(4,245)_ = 4.23, *p* < 0.01], and soma area [*F*_(4,195)_ = 6.19, *p* < 0.0001], the lowest effective dose on dendritic length being 0.1 *μ*M (Figures 5(a)–5(c)). No changes in structural plasticity were observed between basal measurements and measurements after 72 hrs when cultures were exposed to vehicle (Figures 5(a)–5(c), shaded areas). The effects of pramipexole versus vehicle were significantly (*p* < 0.05 or lower, Bonferroni's test) attenuated by pretreatment with SB277011-A (50 nM) and S33084 (10 nM), but not by the D1R antagonist SCH23390 (1 *μ*M) (Figures 5(d)–5(f)), as supported by the significant two-way ANOVA interaction obtained on the maximal length of dendrites [*F*_(43,232)_ = 4.6, *p* < 0.005], number of primary dendrites [*F*_(3,392)_ = 3.7, *p* < 0.02], and soma area [*F*_(3,312)_ = 3.6, *p* < 0.02]. No changes were seen when the DA antagonists were dosed in the presence of vehicle.

The effects of pramipexole on structural plasticity were prevented by inhibition of the intracellular pathways using PD98059 (10 *μ*M), LY294002 (10 *μ*M), and the mTORC1 inhibitor rapamycin (20 nM). All kinase inhibitors significantly (*p* < 0.05 or lower, Bonferroni's test) counteracted the effects of pramipexole on all three structural plasticity parameters (Figures 5(g)–5(i)), as supported by the significant two-way ANOVA interaction obtained on the maximal length of dendrites [*F*_(3,232)_ = 3.1, *p* < 0.05], number of primary dendrites [*F*_(3,392)_ = 4.3, *p* < 0.005], and soma area [*F*_(3,312)_ = 3.8, *p* < 0.01]. No changes were seen when the kinase inhibitors were dosed in the presence of vehicle.

### 3.7. Correlation between the Results Obtained with the Two Human iPSC Clones

Correlation analysis between the mean values of all experiments performed in the DA neurons from the two hiPSC clones (d.f. = 38) was strong and highly significant for the maximal length of dendrites (*r* = 0.872, *p* < 0.001), number of primary dendrites (*r* = 0.966, *p* < 0.001), and soma area (*r* = 0.891, *p* < 0.001).

## 4. Discussion

In the present investigation, the D3R-preferential DA agonists ropinirole and pramipexole produced dose-dependent increases of dendrite outgrowth and soma area of DA neurons derived from human iPSCs. These effects were mediated through BDNF-TrkB and mTOR signaling, key mechanisms known to control dendritic complexity and soma size [30, 47, 48], of which the functional status is impaired in mood disorders [33, 49] and Parkinson's disease [50].

The pharmacological responses of human DA neurons differentiated from the iPSCs of two healthy donors essentially overlapped and were highly correlated. The influence of ropinirole upon structural plasticity in these human DA neurons was similar to that seen in mouse primary DA neurons, the main difference being a systematically larger degree of dendritic arborization and soma size in the former. The present data are well aligned with evidence that D3R-preferential DA agonists, such as 7-OH-DPAT and quinpirole, elicit structural plasticity in rodent DA neurons, effects blocked by D3R-selective antagonists *in vitro* [16] and in *vivo* [27] and absent in D3R knockout mice [16, 28]. In the present investigation, we exposed hiPSC-derived DA neurons to low concentrations of ropinirole and pramipexole, allowing the higher affinity for D3R to exert a preferential role versus D2R [16]; we likewise showed blockade of ropinirole- and pramipexole-induced structural plasticity by preincubation with the highly selective D3R antagonists SB277011-A and S33084.

Ropinirole produced a rapid and D3R-dependent activation (within 2 min) of mTOR signaling in DA neurons as measured by Western blot and immunofluorescence quantification of p-p70S6K. Recruitment of mTOR signaling was blocked by pretreatment with SB277011-A or S33084. In line with these observations, transient increases of phosphorylation in the PI3K-Akt-mTOR pathway were previously observed in mouse DA neurons with agents that directly or indirectly activated D3R [28, 51]. In the present study, we showed that intracellular kinase inhibitors known to block the mTOR signaling, such as rapamycin or LY294002, abrogated both the p70S6K transient phosphorylation and the long-term structural plasticity. Similar results were obtained with the MEK inhibitor PD98059, suggesting crosstalk between Ras-ERK and PI3K-mTOR pathways, as described in other cellular systems [30]. The critical role of D3R is also supported by observations in D3R KO mice in which DA agonists are unable to increase p70S6K phosphorylation and produce long-term structural plasticity [28]. The difference between transient and long-term effects would be that ropinirole and pramipexole trigger transient activation of p70S6K over a constrained time frame that leads to longer-term downstream-sustained processes, such as protein synthesis, necessary for structural plasticity changes. A similar time-restricted phenomenon was described for LTP in rat hippocampal preparations [52]. In this work, the increase of p70S6K phosphorylation observed in neurons during the LTP induction (tetanization) was blocked by brief exposures to mTOR pathway inhibitors only before induction, but not afterward, when LTP was already established.

Since Ras-ERK and PI3K-mTOR pathways are also constitutive elements of the BDNF-TrkB signaling, we explored their role in structural plasticity produced by ropinirole. The application of four different modalities of BDNF-TrkB pathway disruption resulted in structural plasticity blockade in human DA neurons, as previously described in rat telencephalic neurons [46, 48]. These data indicate that active BDNF-TrkB signaling is necessary for D3R-dependent structural plasticity in human DA neurons. Interestingly, the behavioural relevance of reciprocal crosstalk between these two crucial pathways in DA neurons was recently demonstrated in rats with a unilateral nigrostriatal lesion of DA projections [53]. It must be pointed that the degree and duration of enhanced BDNF expression are critical factors since the excessive and prolonged increase of BDNF-TrkB signaling in rodent VTA has been linked to vulnerability to stress [54]. However, other preclinical studies showed an association between low levels of BDNF in VTA with anhedonia [55], while patients with mood disorders were characterized by low TrkB expression in postmortem striatum [56] and low circulating levels of BDNF [49]. Indeed, increased BDNF signaling was recognized as a necessary step for the antidepressant effects of ketamine [57]. Hence, BDNF-TrkB signaling may play a critical role in the long-term structural plasticity of D3-preferential agonists in human DA neurons, possibly contributing to their clinical antidepressant effects so far described for telencephalic neurons [33, 58]. An involvement of D3R in mood disorders is further supported by recent positive trials in insufficiently responsive patients with mood disorders treated with cariprazine, a D2R/D3R partial agonist with a 10-fold preferential affinity to D3R [59, 60]. The intrinsic activity of cariprazine at D3R (Emax 70%) is comparable to that of aripiprazole, another D2R/D3R partial agonist that is approved for the adjunctive treatment of major depression [61]. As recently suggested, both cariprazine and aripiprazole may exert antidepressant effects via D3R-mediated recruitment of BDNF-TrkB signaling [62, 63]. However, a role of their serotonergic properties and postsynaptic D2R influence on depressed states should not be neglected [60, 61].

A limitation of the present study is the use of hiPSCs from healthy donors. Further studies in DA neurons differentiated from hiPSCs derived from patients affected by mood disorders would be of particular interest for enhancing our understanding of how DA agonists influence neuroplasticity under pathological conditions and whether they may be able to restore putative deficits. In this article, we chose to focus on DA neurons from healthy donors in order to systematically characterize the pharmacologic response of clinically active drugs that were previously tested in rodents so to validate the approach.

In conclusion, the present investigation demonstrates that ropinirole and pramipexole increase structural plasticity in human iPSC-derived DA neurons via D3R-dependent mechanisms and recruitment of BDNF-TrkB and mTOR signaling.

These pharmacological/biological mechanisms were remarkably conserved between mouse and human, the only observed difference between species being the neuronal size.

These data support the translational use of human iPSC-derived DA neurons as a possible experimental model for the assessment of pharmacologic agents targeting disorders characterized by a disruption of dopaminergic transmission.

## Figures and Tables

**Table 1:** Statistical analysis of the effects produced by D3R antagonists, by MEK-ERK and PI3K-mTOR inhibitors, and by BDNF-TrkB signaling inhibitors on ropinirole-induced structural plasticity in F3 DA neurons.

Experiments	Two-way ANOVA	Max dendrite length	Number primary dendrites	Soma area
(DA antag.)^1^ X (Rop/Veh)	Interaction	*F*_(4,290)_ = 5.4^∗∗∗^	*F*_(4,490)_ = 4.9^∗∗∗^	*F*_(4,390)_ = 4.3^∗∗^
	Antagonist factor	*F*_(4,290)_ = 6.8^∗∗∗^	*F*_(4,490)_ = 2.8^∗^	*F*_(4,390)_ = 3.8^∗∗^
	Ropinirole factor	*F*_(1,290)_ = 7.6^∗∗^	*F*_(1,490)_ = 7.2^∗∗^	*F*_(1,390)_ = 7.7^∗∗^

(mTOR inh.)^2^ X (Rop/Veh)	Interaction	*F*_(3,232)_ = 3.5^∗^	*F*_(3,392)_ = 4.9^∗∗^	*F*_(3,312)_ = 2.9^∗^
	Inhibitor factor	*F*_(3,232)_ = 4.5^∗∗^	*F*_(3,392)_ = 3.7^∗^	*F*_(3,312)_ = 4^∗∗^
	Ropinirole factor	*F*_(1,232)_ = 7.7^∗∗^	*F*_(1,392)_ = 4^∗^	*F*_(1,312)_ = 6.1^∗^

(TrkB inh.)^3^ X (Rop/Veh)	Interaction	*F*_(4,290)_ = 3.5^∗^	*F*_(4,490)_ = 5.4^∗∗∗^	*F*_(4,390)_ = 2.9^∗^
	Inhibitor factor	*F*_(4,290)_ = 6.3^∗∗∗^	*F*_(4,490)_ = 2.8^∗^	*F*_(4,390)_ = 4.1^∗∗^
	Ropinirole factor	*F*_(1,290)_ = 5.7^∗^	*F*_(1,490)_ = 4.4^∗^	*F*_(1,390)_ = 10^∗∗^

^1^DA antagonists: sulpiride, SB277011-A, S33084, and SCH23390. ^2^mTOR inhibitors: PD98059, LY294002, and rapamycin. ^3^TrkB inhibitors: *α*-BDNF, TrkB-Fc Chimera, K252a, and PP2. The original values represented as mean ± SEM can be found in Figure 4. Two way ANOVA: ^∗∗∗^ *p* < 0.001; ^∗∗^ *p* < 0.01; and ^∗^ *p* < 0.05.

## Abbreviations

7-OH-DPAT: 7-Hydroxy-N,N-di-propyl-2-aminotetralin
Akt: Thymoma viral proto-oncogene
BDNF: Brain-derived neurotrophic factor
DAT: Dopamine transporter
E: Embryonic day
ERK: Extracellular signal-regulated kinase
mTORC1: Mammalian target of rapamycin complex 1
p70S6K: p70 ribosomal S6 protein kinase
p-p70S6K: Phosphorylated p70 ribosomal S6 protein kinase
PI3-K: Phosphatidylinositol-3-kinase
TH: Tyrosine hydroxylase.

## Supplementary Materials

Supplementary Materials Supplementary Table S1: pharmacological agents used in this study. Supplementary Table S2: primary antibodies used in this study. Supplementary Table S3: secondary antibodies used in this study. Supplementary Table S4: primers used in this study. Supplementary Table S5: statistical analysis of the effects produced by D3R antagonists, by MEK-ERK and PI3K-mTOR inhibitors, and by BDNF-TrkB signaling inhibitors on ropinirole-induced structural plasticity in NAS2 DA neurons. Supplementary Figure S1: generation and characterization of F3 hiPSCs. Supplementary Figure S2: TaqMan hPSC Scorecard™ Panel evaluation of F3 hiPSC pluripotency and F3 EB potential for the three germ lineages. Supplementary Figure S3: characterization of NAS2 human iPSCs and NAS2 DA cultures. Supplementary Figure S4: ropinirole increases phosphorylation of p70S6K in NAS2 hiPSC-derived DA neurons via activation of D3 receptor. Supplementary Figure S5: structural plasticity induced by ropinirole in NAS2 DA neurons is prevented by D3R antagonists, by MEK-ERK and PI3K-mTOR inhibitors, and by BDNF-TrkB signaling inhibitors.
