# Supplementary material for: Ropinirole and Pramipexole Promote Structural Plasticity in Human iPSC-Derived Dopaminergic Neurons via BDNF and mTOR Signaling
Source: Neural Plast. 2018 Feb 4;2018:4196961. doi: 10.1155/2018/4196961 (PMC5817382; doi:10.1155/2018/4196961)
Supplement: Supplementary Materials — Supplementary Table S1: pharmacological agents used in this study. Supplementary Table S2: primary antibodies used in this study. Supplementary Table S3: secondary antibodies used in this study. Supplementary Table S4: primers used in this study. Supplementary Table S5: statistical analysis of the effects produced by D3R antagonists, by MEK-ERK and PI3K-mTOR inhibitors, and by BDNF-TrkB signaling inhibitors on ropinirole-induced structural plasticity in NAS2 DA neurons. Supplementary Figure S1: generation and characterization of F3 hiPSCs. Supplementary Figure S2: TaqMan hPSC Scorecard™ Panel evaluation of F3 hiPSC pluripotency and F3 EB potential for the three germ lineages. Supplementary Figure S3: characterization of NAS2 human iPSCs and NAS2 DA cultures. Supplementary Figure S4: ropinirole increases phosphorylation of p70S6K in NAS2 hiPSC-derived DA neurons via activation of D3 receptor. Supplementary Figure S5: structural plasticity induced by ropinirole in NAS2 DA neurons is prevented by D3R antagonists, by MEK-ERK and PI3K-mTOR inhibitors, and by BDNF-TrkB signaling inhibitors. [file 4196961.f1.pdf]

Supplementary Materials

Ropinirole and pramipexole promote structural plasticity in human iPSC-derived dopaminergic neurons *via* BDNF and mTOR signaling

Ginetta Collo, Laura Cavalleri, Federica Bono, Cristina Mora, Stefania Fedele, Roberto William Invernizzi, Massimo Gennarelli, Giovanna Piovani, Tilo Kunath, Mark J. Millan, Emilio Merlo Pich, PierFranco Spano

Supplementary Materials and Methods

Human iPSC generation and characterization

Lentivirus production

For lentivirus generation, 293T cells (System Biosciences, Palo Alto, CA) were transfected with Doxycycline (Dox)-inducible lentiviral vectors for the four Yamanaka's factors. The constructs used were a gift from Konrad Hochedlinger: FU-tet-o-hOct4 (Addgene plasmid #19778), FU-tet-o-hSox2 (Addgene plasmid #19779), FU-tet-o-hKLF4 (Addgene plasmid #19777), FU-tet-o-hc-myc (Addgene plasmid #19775) and the vector for the reverse tetracycline transactivator (FUdeltaGW-rtTA) (Addgene plasmid #19780). Vectors for the packaging constructs (pMDLg/pRRE, pRSV-Rev, pMD2.G) were provided by Addgene (Cambridge, MA). Cells were incubated at 37°C in 10% CO_2. Viruses were harvested over the following 3 days and resuspended in DMEM to obtain a 500-fold concentration (Maherali et al. 2008).

Human fibroblasts reprogramming and iPSC culture

CP01 human fibroblasts were seeded at $1x10^5$ cells in a 35 mm dish 1 day before transduction. For a standard infection, the medium was replaced with Dox-inducible lentiviruses: 5 μl of each factor (Oct4, Sox2, KLF4), 2 μl of c-myc, 10 μl of rtTA, supplemented with 6 μg/ml of polybrene (Sigma-Aldrich) and incubated overnight (Maherali et al. 2008) (Supplementary Fig. S1A). Two days after transduction, infected fibroblasts were replated on irradiated mouse embryonic fibroblasts (MEF) feeders (MTI-GlobalStem, Gaithersburg, MD) in hESC medium containing Knockout™ DMEM, 10% KSR, non-essential amino acids, GlutaMAX™ and 55 μM 2-mercaptoethanol (all from Gibco-Invitrogen) supplemented with 10 ng/ml recombinant human basic fibroblast growth factor (bFGF; R&D Systems,

Minneapolis, MN), 1 μg/ml Dox (Sigma-Aldrich) and 2% FBS. From day 10 Dox was progressively reduced and one week after complete removal four emerging colonies were identified, picked, mechanically dissociated and transferred on MEF feeders (Maherali et al. 2008) (Supplementary Fig. S1A). Two colonies that maintained the hESC-like characteristics were selected. In this study, the clone CP01-F3 (F3) was used. F3 human iPSCs at passage 13th on MEF feeders were adapted to feeder-free conditions on Matrigel-coated plates (BD Matrigel hESC qualified-Matrix, BD Biosciences, San Jose, CA) and mTeSR™1 medium (StemCell Technologies, Vancouver, BC, Canada) supplemented with ROCK inhibitor (Y-27632 10 μM; Tocris Bioscience).

NAS2 human iPSCs (Devine et al. 2011) were cultured on Matrigel in mTeSR™1 medium.

Embryoid bodies generation and differentiation

Human iPSCs were mechanically dissociated and placed in suspension culture in hESC medium. After 8 days embryoid bodies (EBs) formed. At this stage EBs were plated into adherent conditions on Matrigel-coated dishes (for RNA extraction) or coverslides (for immunostaining) and maintained in culture 8 days to induce spontaneous differentiation.

Alkaline Phosphatase staining of hiPSCs

For the detection of Alkaline Phosphatase (AP), F3 human iPSCs were fixed with PBS containing 3% paraformaldehyde (2 min at RT) and incubated with Fast Red Violet Solution, Napthol AS-BI Phosphate Solution (all from Merck Millipore, Milan, Italy) and water in a 2:1:1 ratio. The samples were visualized using an Olympus IX51 microscope.

TaqMan Scorecard analysis of human iPSCs and embryoid bodies

Expression of pluripotency markers and determination of trilineage differentiation potential were evaluated for F3 hiPSCs and randomly differentiated cells from F3 EBs using the TaqMan hPSC Scorecard^{TM} Panel kit (96 well, StepOne Plus; Thermo Fisher Scientific, MA) following the company's guidelines and using its cloud-based online analysis software. Total RNA was isolated using Quick-RNA MiniPrep (Zymo Research). Single-strand cDNA was obtained from 1 μg of total RNA by reverse transcription (RT) using the high-capacity cDNA RT Kit (Thermo Fisher Scientific). cDNA samples were analyzed in duplicate by qRT-PCR using the TaqMan hPSC Scorecard Panel. Pluripotency of the F3 hiPSCs and their trilineage differentiation potential was determined by comparing mean cycle threshold (Ct) values obtained for each marker to the values of the reference standards (represented by nine undifferentiated pluripotent stem cell lines, including human embryonic stem cells and iPSCs). A proprietary algorithm (Thermo Fisher Scientific) calculated the relative score for iPSCs based on how well the expression of each gene correlates with reference lines (Ferrer et al. 2014).

Karyotype analysis

Cytogenetic studies were performed on chromosomes from CP01 human fibroblasts and from F3 hiPSCs after seven passages. Chromosome preparations were obtained according to standard techniques for cytogenetic samples adding 10 μg/ml Colcemid Solution (Irvine Scientific, Santa Ana, CA) in Hank's balanced salt solution (Sigma-Aldrich). 10-20 colonies were picked dissociated using Trypsin / EDTA (Sigma-Aldrich). Cells were incubated in hypotonic solution and then fixed in methanol and

acetic acid (3:1). Metaphase suitable for analysis was sequentially Q-banded according to the routine methods.

Immunofluorescence and immunocytochemistry

For immunofluorescence, F3 hiPSCs, F3 EBs and hiPSC-derived DA neurons were fixed with either 3% paraformaldehyde in 3% sucrose / PBS (20 min at RT) or 100% methanol (10 min at -20°C), blocked and permeabilized with PBS, 0.2% Triton, 1% normal goat serum, 5% bovine serum albumin (30 min at RT) and incubated overnight at 4°C with primary antibodies (Supplementary Table S2). Appropriate Alexa Fluor® 488- and Cy™3-conjugated secondary antibodies (Jackson ImmunoResearch) (Supplementary Table S3) were incubated 1 hr at RT, followed by DAPI (Molecular Probes-Invitrogen). Each experiment was repeated three times. The samples were visualized using a Zeiss Axio Observer Z1 completed with ApoTome.2 (Carl Zeiss AG, Oberkochen, Germany) or Olympus IX51 microscope (Olympus Italia Srl, Milano, Italy).

RNA extraction and RT-PCR analysis

Total RNA from hiPSC-derived DA cultures was extracted using Quick-RNA MiniPrep (Zymo Research, Irvine, CA) according to the manufacturer's instructions. RNA quantification and quality controls were performed using spectrophotometric analysis (NanoDrop Technologies, Wilmington, DE). Reverse transcription was carried out using Moloney Murine Leukemia Virus-Reverse Transcriptase (MMLV-RT; Invitrogen). RNA expression pattern of target genes was studied with the DreamTAQ Green PCR Master Mix (2X) (Thermo Scientific, Milan, Italy) following the manufacturer's instructions. PCR products were visualized by agarose/ethidium bromide gel electrophoresis.

Supplementary Results

Characterization of the F3 human iPSC clone

F3 hiPSC clone was selected out of four clones resulting from the reprogramming of CP01 fibroblasts (Supplementary Fig. S1A-D). F3 hiPSCs strongly expressed Alkaline Phosphatase (AP) and were positive for the pluripotency markers Oct3/4, Sox2, Nanog, TRA-1-60 and TRA-1-81 by immunocytochemistry (Supplementary Fig. S1E-J), semi-quantitative RT-PCR confirmed the expression of pluripotency genes Oct3/4, Sox2 and Nanog (Supplementary Fig. S1K). Analysis of the karyotype of the fibroblasts used for the reprogramming and of the derived F3 hiPSCs over several passages showed no chromosomal aberrations (Supplementary Fig. S1L). F3 hiPSCs at passage 13 were transferred to feeder free conditions to be used in the subsequent experiments (Supplementary Fig. S1M). Their pluripotency potential was examined *in vitro* by EBs formation (Supplementary Fig. S1N) and differentiation. After 8 days in suspension culture, EBs were transferred to adherent conditions for 8 more days giving rise to cells of the three germ layer lineages. Immunofluorescence analysis showed endodermal cells positive for α-fetoprotein (AFP) (Supplementary Fig. S1O), mesodermal cells positive for smooth muscle actin (SMA) and brachyury (Supplementary Fig. S1P-Q) and ectodermal cells positive for βIII Tubulin and GFAP (Supplementary Fig. S1R-S). The pluripotency of F3 hiPSCs and their differentiation potential into any of the three germ layers was studied using the TaqMan hPSC Scorecard^{TM} Panel kit (Supplementary Fig. S2A-D) (Ferrer et al. 2014; Tsankov et al. 2015). The pluripotency marker expression of F3 hiPSCs at passage 23 was comparable to the reference standard. With regard to germ layer markers, F3 hiPSCs at passage 23 showed downregulated expression values for endoderm markers (-1.14

folds) and ectoderm markers (-0.84 folds) compared to the reference standard. Mesoderm markers were comparable to the reference standard.

Supplementary Table S1. Pharmacological agents used in this study.

Name	Supplier	Catalog number	Solvent	Final concentration
α-BDNF [1]	Merck Millipore	AB1513P	Water	10 µg/ml
GBR12935 [2]	Sigma-Aldrich	G9659	Water	0.03 µM
K252a [3]	Tocris Bioscience	1683	DMSO	0.2 µM
LY294002 [4]	Tocris Bioscience	1130	DMSO	10 µM
PD98059 [5]	Tocris Bioscience	1213	DMSO	10 µM
PP2 [6]	Tocris Bioscience	1407	DMSO	10 µM
Pramipexole [7]	Tocris Bioscience	4174	DMSO	0.01-10 µM
Rapamycin [8]	Tocris Bioscience	1292	DMSO	0.02 µM
Ropinirole [9]	Tocris Bioscience	3680	Water	0.1-20 µM
S33084 [10]	Institut de Recherches Servier	/	DMSO	0.01 µM
SB277011-A [11]	Tocris Bioscience	4207	DMSO	0.05 µM
SCH23390 [12]	Tocris Bioscience	0925	Water	1 µM
Sulpiride [13]	Tocris Bioscience	0895	Ethanol	5 µM
TrkB-Fc Chimera [14]	R&D Systems	688-TK	PBS	5 µg/ml

[1] Anti-Brain Derived Neurotrophic Factor antibody; [2] 1-(2-Benzhydryloxyethyl)-4-(3-phenylpropyl)piperazine dihydrochloride; [3] (9S,10R,12R)-2,3,9,10,11,12-Hexahydro-10-hydroxy-9-methyl-1-oxo-9,12-epoxy-1H-diindolo[1,2,3-fg:3',2',1'-kl]pyrrolo[3,4-i][1,6]benzodiazocine-10-carboxylic acid methyl ester; [4] 2-(4-Morpholinyl)-8-phenyl-4H-1-benzopyran-4-one hydrochloride; [5] 2-(2-Amino-3-methoxyphenyl)-4H-1-benzopyran-4-one; [6] 3-(4-Chlorophenyl) 1-(1,1-dimethylethyl)-1H-pyrazolo[3,4-d]pyrimidin-4-amine; [7] 2-Amino-4,5,6,7-tetrahydro-6-(propylamino)benzothiazole dihydrochloride; [8](3S,6R,7E,9R,10R,12R,14S,15E,17E,19E,21S,23S,26R,27R,34aS)-9,10,12,13,14,21,22,23,24,25,26,27,32,33,34,34a-Hexadecahydro-9,27-dihydroxy-3-[(1R)-2-[(1S,3R,4R)-4-hydroxy-3-methoxycyclohexyl]-1-methylethyl]-10,21-dimethoxy-6,8,12,14,20,26-hexamethyl-23,27-epoxy-3H-pyrido[2,1-c][1,4]oxaazacyclohentriacontine-1,5,11,28,29(4H,6H,31H)-pentone; [9] 4-[2-(Dipropylamino)ethyl]-1,3-dihydro-2H-indol-2-one hydrochloride; [10] N-[4-[(3aR,9bS)-8-Cyano-3,3a,4,9b-tetrahydro-1H-chromeno[3,4-c]pyrrol-2-yl]butyl]-4-phenylbenzamide; [11] N-[trans-4-[2-(6-Cyano-3,4-dihydro-2(1H)-isoquinolinyl)ethyl]cyclohexyl]-4-quinolinecarboxamide dihydrochloride; [12] (R)-(+)-7-Chloro-8-hydroxy-3-methyl-1-phenyl-2,3,4,5-tetrahydro-1H-3-benzazepine hydrochloride; [13] (S)-(-)-5-Aminosulfonyl-N-[(1-ethyl-2-pyrrolidinyl)methyl]-2-methoxybenzamide; [14] Recombinant Human Tyrosine Kinase Receptor B Fc Chimera Protein.

Supplementary Table S2. Primary antibodies used in this study.

Name	Supplier	Catalog number	Species	Final dilution	Detection method
α-Tubulin	Sigma-Aldrich	T5168	Mouse mAb	1:20000	WB
βIII Tubulin	Sigma-Aldrich	T8660	Mouse mAb	1:600	IF
AFP	R&D Systems	MAB1368	Mouse mAb	1:100	IF
Brachyury	R&D Systems	AF2085	Goat pAb	1:100	IF
DAT	Santa Cruz Biotechnology	sc-32258	Rat mAb	1:400	IF
HNF-3β (FoxA2)	Santa Cruz Biotechnology	sc-9187	Goat pAb	1:500	IF
GAD67	Merck Millipore	MAB5406	Mouse mAb	1:700	IF
GFAP	Sigma-Aldrich	G3893	Mouse mAb	1:100	IF
GLUR2	Alomone Labs	AGC-005	Rabbit pAb	1:200	IF
LMX1A	Sigma-Aldrich	HPA030088	Rabbit pAb	1:1000	IF
MAP2	Merck Millipore	AB5622	Rabbit pAb	1:1000	IF
Nanog	Merck Millipore	MABD24	Mouse mAb	1:200	IF
Oct3/4	Merck Millipore	MAB4419	Mouse mAb	1:200	IF
p70S6K	Cell Signaling Technology	2708	Rabbit mAb	1:4000	WB
p-p70S6K$^{(Thr389)}$	Cell Signaling Technology	9206	Mouse mAb	1:500	IF
				1:4000	WB
SMA	Dako	M0851	Mouse mAb	1:200	IF
Sox2	Merck Millipore	MAB4423	Mouse mAb	1:200	IF
TH	Merck Millipore	MAB318	Mouse mAb	1:500	IF
TH	Merck Millipore	AB152	Rabbit pAb	1:4000	WB
TH	Santa Cruz Biotechnology	sc-14007	Rabbit pAb	1:500	IF, IHC
TRA-1-60	Merck Millipore	MAB4360	Mouse mAb	1:200	IF
TRA-1-81	Merck Millipore	MAB4381	Mouse mAb	1:200	IF
VGLUT2	Merck Millipore	MAB5504	Mouse mAb	1:500	IF
VMAT2	Merck Millipore	AB1598P	Rabbit pAb	1:50	IF

Supplementary Table S3. Secondary antibodies used in this study.

Name	Supplier	Catalog number	Final dilution	Detection method
Biotinylated goat anti-rabbit IgG	Vector Laboratories	BA-1000	1:350	ICC
Cy™3-conjugated goat anti-mouse IgG	Jackson ImmunoResearch	115-166-062	1:800	IF
Cy™3-conjugated goat anti-rabbit IgG	Jackson ImmunoResearch	111-166-045	1:1000	IF
Cy™3-conjugated goat anti-rat IgG	Jackson ImmunoResearch	112-165-167	1:1000	IF
Alexa Fluor® 488-conjugated goat anti-mouse IgG	Jackson ImmunoResearch	115-546-071	1:500	IF
Alexa Fluor® 488-conjugated goat anti-rabbit IgG	Jackson ImmunoResearch	111-485-144	1:500	IF
HRP-conjugated goat anti-mouse IgG	Santa Cruz Biotechnology	sc-2005	1:5000	WB
HRP-conjugated goat anti-rabbit IgG	Santa Cruz Biotechnology	sc-2030	1:5000	WB

Supplementary Table S4. Primers used in this study.

Primers	RefSeq	Primers sequence	Primers length	Tm
β-actin F	NG_007992.1 on chr 7	GAAGAGCTACGAGCTGCCTGA	21	60°C
β-actin R	NM_001101.3 mRNA	TGATCTTCATTGTGCTGGGTG	21	
DDC F	XM_005271745.2	CGCCAGGATCCCCGCTTTGAAATCTG	26	68°C
DDC R		TCGGCCGCCAGCTCTTTGATGTGTTC	26	
D2R F		GCAGACCACCACCAACTACC	20	58°C
D2R R		GGAGCTGTAGCGCGTATTGT	20	
D3R F		TGGCTGCAGGAGCCGAAGT	19	68°C
D3R R		GAGGGCAGGACACAGCAAAGGC	22	
EN1 F	NM_001426.3	GCAACCCGGCTATCCTACTTATG	23	60°C
EN1 R		ATGTAGCGGTTTGCCTGGAAC	21	
FOXA2 F	NM_021784.4	CTGGGAGCGGTGAAGATGGA	20	62°C
FOXA2 R	NM_153675.2	ACGTACGACGACATGTTCATGGAG	24	
GAPDH F		GCTCAGACACCATGGGGAAGGT	22	68°C
GAPDH R		GTGGTGCAGGAGGCATTGCTGA	22	
GIRK2 F		GCTACCGGGTCATCACAGAT	20	60°C
GIRK2 R		ACTGCATGGGTGGAAAAGAC	20	
LMX1A F	NM_177398.3	CAGCCTCAGACTCAGGTAAAAGTG	24	60°C
LMX1A R		TGAATGCTCGCCTCTGTTGA	20	
LMX1B F	NM_002316.3	ACGAGGAGTGTTTGCAGTGCG	21	62°C
LMX1B R		CCCTCCTTGAGCACGAATTCG	21	
Nanog F	XM_005253484.2	CAGCCCTGATTCTTCCACCAGTCCC	25	68°C
Nanog R	NM_024865.2	TGGAAGGTTCCCAGTCGGGTTCACC	25	
NURR1 F	NM_006186.3	CTATTCCAGGTTCCAGGCGAA	21	60°C
NURR1 R		CTGGGTTGGACCTGTATGCTAA	22	
Oct3/4 F	NM_001285987.1	GACAGGGGGAGGGGAGGAGCTAGG	24	68°C
Oct3/4 R		CTTCCCTCCAACCAGTTGCCCCAAAC	26	
Sox2 F	NM_003106.3	GGGAAATGGGAGGGGTGCAAAAGAGG	26	68°C
Sox2 R		TTGCGTGAGTGTGGATGGGATTGGTG	26	
TH F	NM_199293.2	GTCCCCTGGTTCCCAAGAAAAGT	23	62°C
TH R		TCCAGCTGGGGGATATTGTCTTC	23	

Supplementary Table S5. Statistical analysis of the effects produced by D3R antagonists, by MEK-ERK and PI3K-mTOR inhibitors and by BDNF-TrkB signaling inhibitors on ropinirole-induced structural plasticity in NAS2 DA neurons.

Experiments	Two-way ANOVA	Max dendrite length	Number primary dendrites	Soma area
(DA antag.) [1]	Interaction	F(4,290) = 4.1 **	F(4,490) = 4.3 **	F(4,380) = 3.6 **
X	Antagonist Factor	F(4,290) = 6.7 ****	F(4,490) = 5.6 ***	F(4,380) = 7.7 ****
(Rop/Veh)	Ropinirole Factor	F(1,290) = 10 **	F(1,490) = 5.8 *	F(1,380) = 11 ***
(mTOR inh.) [2]	Interaction	F(3,232) = 11.0 ***	F(3,392) = 6.6 ***	F(3,312) = 5.1 **
X	Inhibitor Factor	F(3,232) = 9.3 ***	F(3, 392) = 3.6 *	F(3, 312) = 9.5 ****
(Rop/Veh)	Ropinirole Factor	F(1,232) = 4.5 *	F(1, 392) = 0.70	F(1, 312) = 4.1*
(TrkB inh.) [3]	Interaction	F(4,290) = 12.0 ***	F(4,490) = 3.5 **	F(4,390) = 7.0 ****
X	Inhibitor Factor	F(4,290) = 3.1 *	F(4,490) = 3.2	F(4,390) = 2.6 *
(Rop/Veh)	Ropinirole Factor	F(1,290) = 3.9 *	F(1,490) = 1.0	F(1,390) = 6.7 *

[1] DA antagonists: Sulpiride, SB277011-A, S33084, SCH23390; [2] mTOR inhibitors: PD98059, LY294002, Rapamycin; [3] TrkB inhibitors: α-BDNF, TrkB-Fc Chimera, K252a, PP2

The original values represented as mean ± S.E.M can be found in Supplementary Figure S5.Two way ANOVA: *** P<0.001; ** P<0.01; * P<0.05

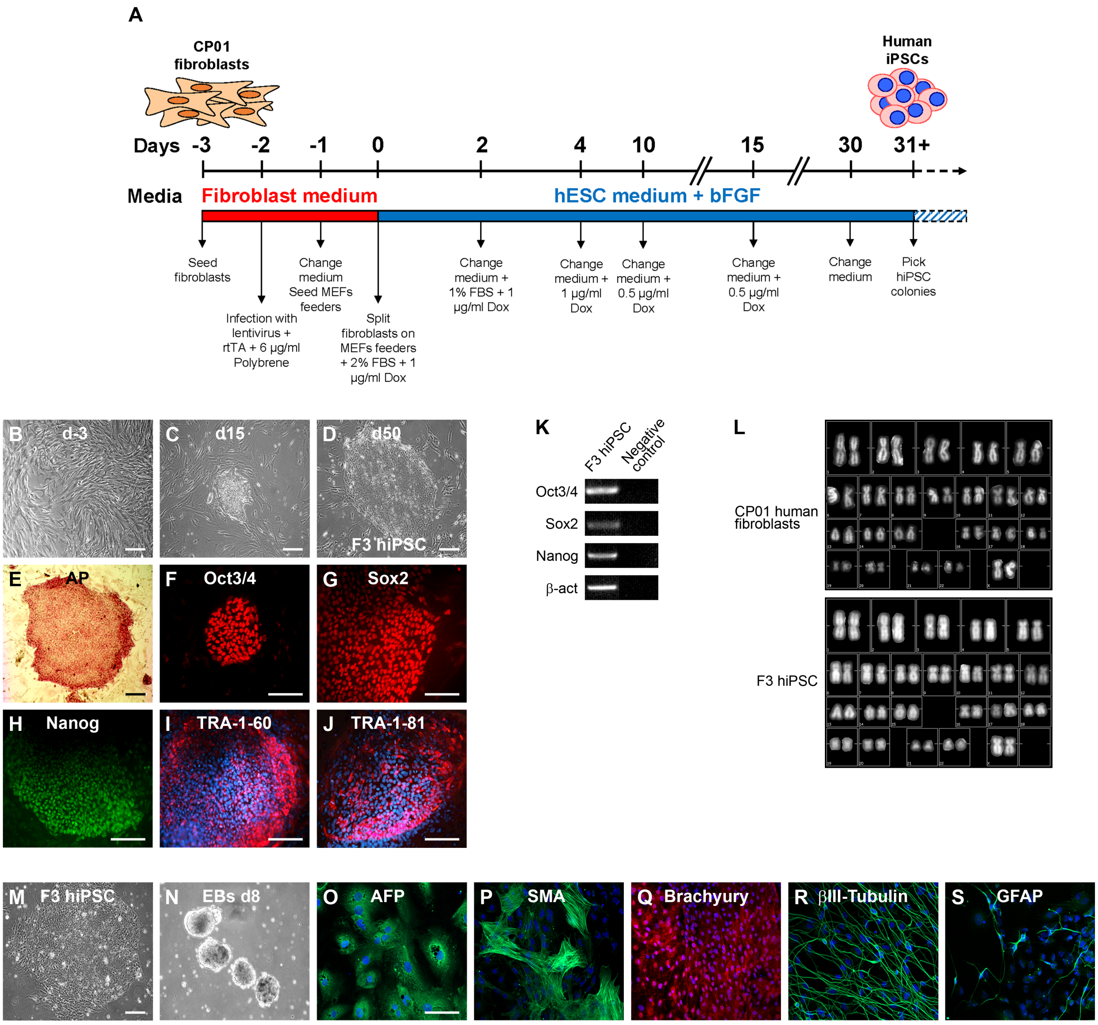

Supplementary Figure S1. Generation and characterization of F3 hiPSCs. (A) Diagram of the time line and culture conditions required for reprogramming CP01 fibroblasts to hiPSCs. **(B-D)** Representative phase contrast images showing the morphology of **(B)** CP01 fibroblasts before reprogramming, **(C)** hiPSC colonies 15 days after reprogramming, **(D)** established F3 hiPSC colonies 50 days after reprogramming. **(E-J)** Representative photomicrographs of F3 hiPSC immunostaining showing the expression of pluripotent markers, **(E)** Alkaline Phosphatase (AP), **(F)** Oct3/4, **(G)** Sox2, **(H)** Nanog, **(I)** TRA-1-60, **(J)** TRA-1-81. **(K)** RT-PCR analysis for pluripotency markers. **(L)** Karyotype of CP01 fibroblasts, before reprogramming and of F3 hiPSCs after 7 passages. **(M)** Representative image of F3 hiPSCs transferred to Matrigel and cultured in feeder-free conditions. **(N)** Embryoid bodies (EBs) after 8 days culture in suspension. **(O-S)** Immunofluorescence analysis of the three germ layer cells differentiated from EBs cultured 8 days in adherent condition: **(O)** Alpha-fetoprotein (AFP), **(P)** smooth muscle actin (SMA), **(Q)** brachyury, **(R)** βIII Tubulin,

(S) glial fibrillary acidic protein (GFAP). Cell nuclei were stained with DAPI (blue). Scale bar: (B-E, M and N) = 40 μm; (F-J, O-S) = 20 μm.

A

Sample Name	Self-renewal	Ectoderm	Mesoderm	Endoderm
F3 hiPSC	0.10	-0.84	-0.03	-1.14

Gene expression relative to the reference standard

Upregulated						Downregulated
x > 1.5	1.0 < x ≤ 1.5	0.5 < x ≤ 1.0	-0.5 ≤ x ≤ 0.5	-1.0 ≤ x < -0.5	-1.5 ≤ x < -1.0	x < -1.5

B

Sample Name	Self-renewal	Ectoderm	Mesoderm	Endoderm
F3 EB	-4.00	1.81	3.14	0.79

Gene expression relative to the reference standard

Upregulated						Downregulated
x > 1.5	1.0 < x ≤ 1.5	0.5 < x ≤ 1.0	-0.5 ≤ x ≤ 0.5	-1.0 ≤ x < -0.5	-1.5 ≤ x < -1.0	x < -1.5

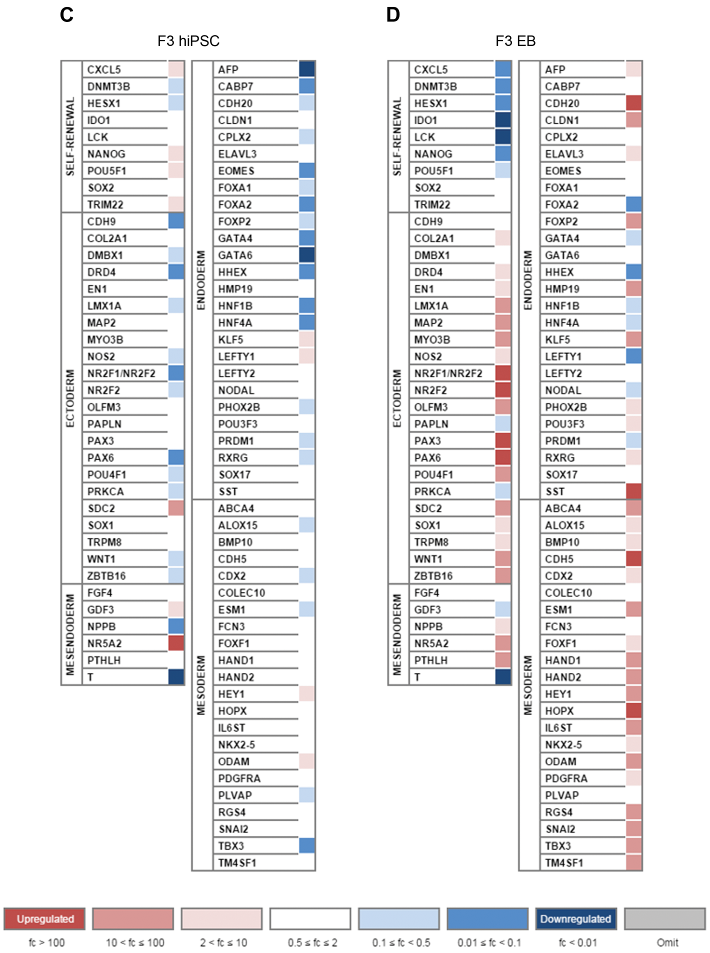

Supplementary Figure S2. TaqMan hPSC Scorecard™ Panel evaluation of F3 hiPSC pluripotency **(A and C)** and F3 EB potential for the three germ lineages **(B and D)**. In **(A and B)** is indicated the gene expression of F3 hiPSCs and F3 EBs respectively, relative to the reference standard. In **(C and D)** are listed the pluripotency genes **(C)** and the genes representative of the three germ layers **(D)** studied using the TaqMan hPSC Scorecard^{TM} Panel kit.

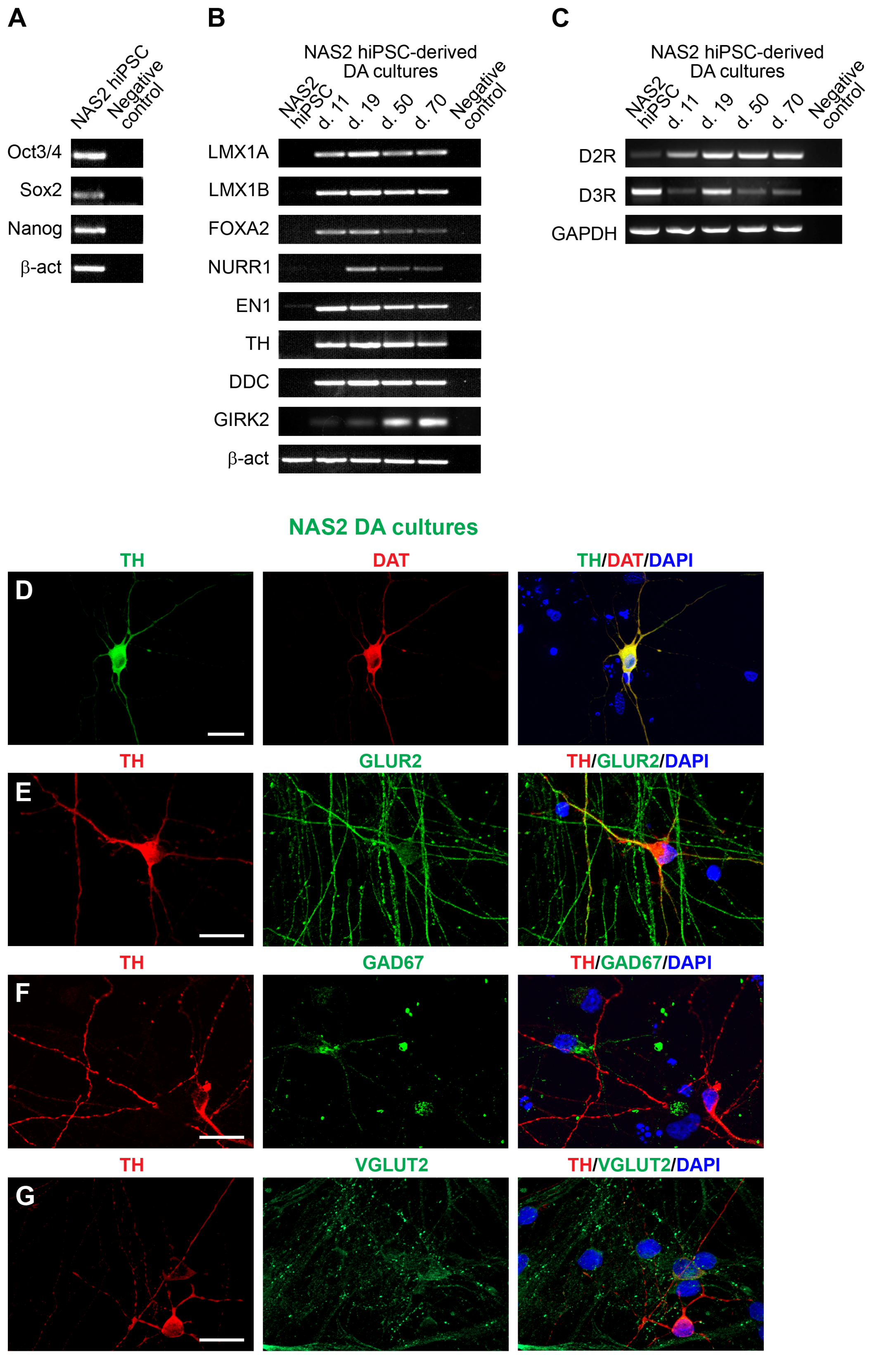

Supplementary Figure S3. Characterization of NAS2 human iPSCs and NAS2 DA cultures. (A) Semi-quantitative RT-PCR analysis of NAS2 hiPSCs for the expression of pluripotency markers Oct3/4, Sox2 and Nanog. **(B and C)** Semi-

quantitative RT-PCR analysis of **(B)** gene expression at the iPSC stage and at day 11, 19, 50 and 70 of dopaminergic differentiation, **(C)** expression of D2 and D3 receptors at the iPSC stage and at day 11, 19, 50 and 70 of dopaminergic differentiation (negative controls contain PCR Master Mix and primers, but no cDNA). **(D-G)** Representative images of dual immunofluorescence at day 70 of **(D)** TH (green), DAT (red) neurons, **(E)** TH (red), GLUR2 (green) neurons, **(F)** TH (red), GAD67 (green) neurons, **(G)** TH (red), VGLUT2 (green) neurons. Cell count indicated 32%±6% TH^+ neurons, 22%±5% $GAD67^+$ neurons and 30%±5% $VGLUT2^+$ neurons co-stained with anti-MAP2 antibody, $MAP2^+$ neurons constituting about 90% of the total number of cells in the culture. Cell nuclei were stained with DAPI (blue). Scale bar: (D) = 30 μm; (E-G) = 20 μm.

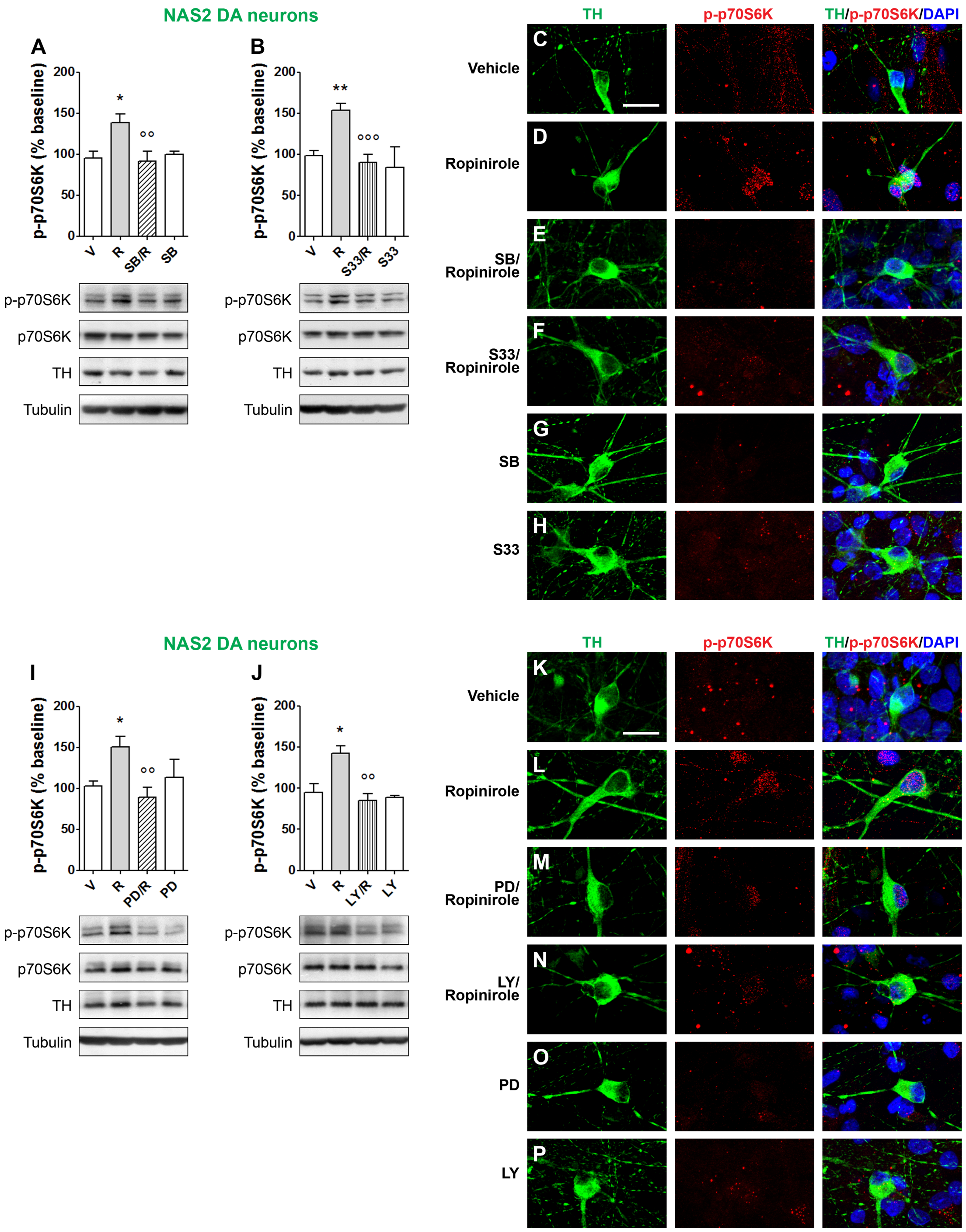

Supplementary Figure S4. Ropinirole increases phosphorylation of p70S6K in NAS2 hiPSC-derived DA neurons via activation of D3 receptor. (A and B) Blockade of p-p70S6K induced by ropinirole (10 μM) in NAS2 DA neurons after pretreatment (20 min) with the D3R antagonists **(A)** SB277011-A (100 nM) or **(B)** S33084 (10 nM) assessed after 2 min and analyzed by densitometry of western blots (n=3) (top panels). Lower panels: representative western blots. **(C-H)** Representative photomicrographs of NAS2 DA neurons showing p-p70S6K increase in the soma and

dendrites of TH$^+$ neurons 2 min after ropinirole (10 μM) following pretreatment with vehicle **(D)**. **(E)** Blockade of p-p70S6K by pretreatment (20 min) with either SB277011-A (100 nM) or **(F)** S33084 (10 nM) followed by ropinirole (2 min). **(C)** Vehicle; **(G)** SB277011-A alone; **(H)** S33084 alone. TH (green), p-p70S6K (red). Cell nuclei were stained with DAPI (blue). (**I and J**) Blockade of p-p70S6K induced by ropinirole (10 μM) in NAS2 DA neurons after pretreatment (20 min) with **(I)** the MEK inhibitor PD98059 (10 μM) or **(J)** PI3-K inhibitor LY294002 (10 μM) assessed after 2 min and analyzed by densitometry of western blots (n=3) (top panels). Lower panels: representative western blots. **(K-P)** Representative photomicrographs of NAS2 DA neurons showing p-p70S6K increase in the soma and dendrites of TH$^+$ neurons 2 min after ropinirole (10 μM) following pretreatment with vehicle **(L)**. **(M)** Blockade of p-p70S6K by pretreatment (20 min) with PD98059 (10 μM) or **(N)** LY294002 (10 μM) followed by ropinirole (2 min). **(K)** Vehicle; **(O)** PD98059 alone; **(P)** LY294002 alone. TH (green), p-p70S6K (red). Cell nuclei were stained with DAPI (blue). Scale bar: 20 μm. All data are expressed as mean values ± S.E.M. (***P*<0.01, **P*<0.05 vs. vehicle; ^{ooo}P<0.001, ^{oo}P<0.01 vs. ropinirole; *post-hoc* Bonferroni's test. Abbreviations: V, vehicle; R, ropinirole; SB, SB277011-A; S33, S33084; PD, PD98059; LY, LY294002.

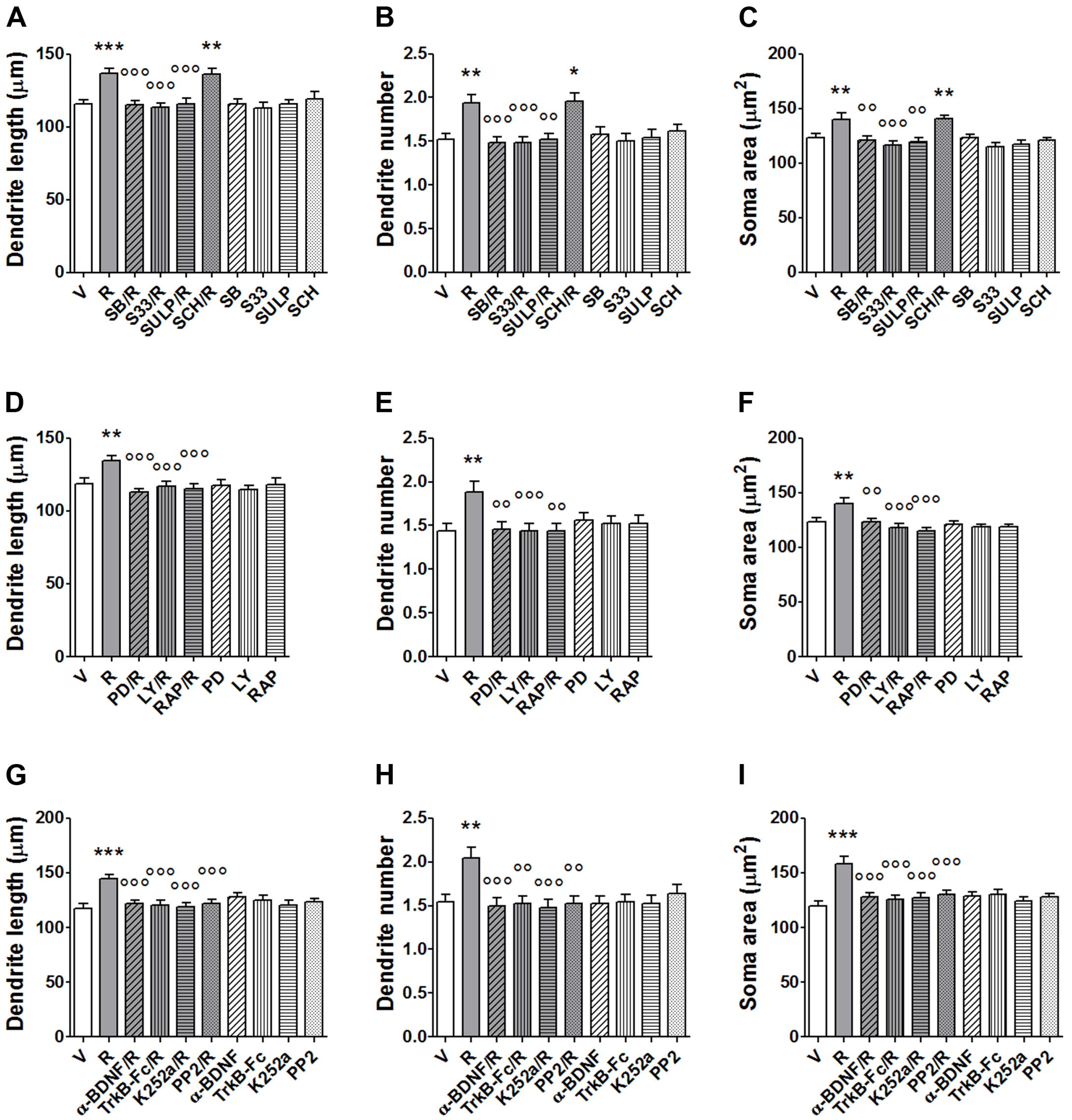

Supplementary Figure S5. Structural plasticity induced by ropinirole in NAS2 DA neurons is prevented by D3R antagonists, by MEK-ERK and PI3K-mTOR inhibitors and by BDNF-TrkB signaling inhibitors. (A-C) Inhibition of the effects of ropinirole (10 μM) on structural plasticity of NAS2 DA neurons following pretreatment (20 min) with the D3R antagonists SB277011-A (50 nM) and S33084 (10 nM) or the D2/D3R antagonist sulpiride (5 μM) assessed as **(A)** maximal dendrite length, **(B)** number of primary dendrites and **(C)** soma area after 72 hrs. Pretreatment (20 min) with the D1R antagonist SCH23390 (1 μM) was ineffective. **(D-F)** Inhibition of the effects of ropinirole (10 μM) on structural plasticity of NAS2 DA neurons following pretreatment (20 min) with PD98059 (10 μM), LY294002 (10 μM) or rapamycin (20 nM), assessed as **(D)** maximal dendrite length, **(E)** number of

primary dendrites and **(F)** soma area after 72 hrs. **(G-I)** Inhibition of the effects of ropinirole (10 μM) on structural plasticity of NAS2 DA neurons following pretreatment (20 min) with an anti-BDNF blocking antibody (α−BDNF) (10 μg/ml), a TrkB-Fc Chimera (5 μg/ml), the TrkB-phosphorylation inhibitor K252a (200 nM) and the TrkB-Src phosphorylation inhibitor PP2 (10 μM), assessed as **(G)** maximal dendrite length, **(H)** number of primary dendrites and **(I)** soma area after 72 hrs. When antagonists and inhibitors were tested with the vehicle, no changes of structural plasticity were visualized. In all panels, values are represented as mean ± S.E.M. (****P*<0.001; ***P*<0.01; **P*<0.05 vs. vehicle; °°°*P*<0.001; °°*P*<0.01 vs. ropinirole; *post-hoc* Bonferroni's test). The results of two-way ANOVA statistical analysis are shown in Supplementary Table S5. Abbreviations: V, vehicle; R, ropinirole; SB, SB277011-A; S33, S33084; SULP, sulpiride; SCH, SCH23390; PD, PD98059; LY, LY294002; RAP, rapamycin. α−BDNF, anti-BDNF blocking antibody; TrkB-Fc, TrkB-Fc Chimera.

Supplementary references

Ferrer M, Corneo B, Davis J, Wan Q, Miyagishima KJ, King R, et al (2014). A multiplex high-throughput gene expression assay to simultaneously detect disease and functional markers in induced pluripotent stem cell-derived retinal pigment epithelium. *Stem Cells Transl Med* **3**:911-922.

Tsankov AM, Akopian V, Pop R, Chetty S, Gifford CA, Daheron L, et al (2015). A qPCR ScoreCard quantifies the differentiation potential of human pluripotent stem cells. *Nat Biotechnol* **33**:1182-1192.
